# Serum inflammatory cytokines as disease biomarkers in the DE50-MD dog model of Duchenne muscular dystrophy

**DOI:** 10.1242/dmm.049394

**Published:** 2022-12-09

**Authors:** Dominique O. Riddell, John C. W. Hildyard, Rachel C. M. Harron, Natasha L. Hornby, Dominic J. Wells, Richard J. Piercy

**Affiliations:** ^1^ Comparative Neuromuscular Diseases Laboratory, Department of Clinical Science and Services, Royal Veterinary College, Camden, London NW1 0TU, UK; ^2^ Department of Comparative Biomedical Sciences, Royal Veterinary College, Camden, London NW1 0TU, UK

**Keywords:** Dystrophin, Duchenne muscular dystrophy, DE50-MD dog model, Inflammatory cytokines, CCL2, Serum biomarker

## Abstract

Duchenne muscular dystrophy (DMD) is a fatal muscle-wasting disease, caused by mutations in the dystrophin gene, characterised by cycles of muscle degeneration, inflammation and regeneration. Recently, there has been renewed interest specifically in drugs that ameliorate muscle inflammation in DMD patients. The DE50-MD dog is a model of DMD that closely mimics the human DMD phenotype. We quantified inflammatory proteins in serum from wild-type (WT) and DE50-MD dogs aged 3-18 months to identify biomarkers for future pre-clinical trials. Significantly higher concentrations of C-C motif chemokine ligand 2 (CCL2), granulocyte-macrophage colony-stimulating factor (GM-CSF or CSF2), keratinocyte chemotactic-like (KC-like, homologous to mouse CXCL1), TNFα (or TNF), and interleukins IL2, IL6, IL7, IL8 (CXCL8), IL10, IL15 and IL18 were detected in DE50-MD serum compared to WT serum. Of these, CCL2 best differentiated the two genotypes. The relative level of CCL2 mRNA was greater in the vastus lateralis muscle of DE50-MD dogs than in that of WT dogs, and CCL2 was expressed both within and at the periphery of damaged myofibres. Serum CCL2 concentration was significantly associated with acid phosphatase staining in vastus lateralis biopsy samples in DE50-MD dogs. In conclusion, the serum cytokine profile suggests that inflammation is a feature of the DE50-MD phenotype. Quantification of serum CCL2 in particular is a useful non-invasive biomarker of the DE50-MD phenotype.

## Teaser

**Summary:** An evaluation of the serum inflammatory cytokine profile of the newly established DE50-MD dog model of Duchenne muscular dystrophy revealed potential biomarkers for future pre-clinical trials.

## INTRODUCTION

Duchenne muscular dystrophy (DMD) is a progressive muscle-wasting disorder caused by mutations in the dystrophin gene. Absence of muscle dystrophin is associated with increased susceptibility to contraction-induced sarcolemmal damage (Duchenne, 1868; Duan et al., 2021). Muscle samples from DMD patients of all ages have areas of inflammation associated with necrotic, degenerating and regenerating fibres (Duan et al., 2021). Although inflammatory cells are necessary for the clearance of degraded muscle tissue, chronic inflammation as a result of continuous muscle necrosis in DMD contributes to muscle weakness by further impairing muscle repair and promoting fibrosis (Wynn, 2008; Shin et al., 2013).

Cytokines released by dystrophic muscles recruit immune cells to infiltrate and migrate through damaged tissue to initiate repair. The inflammatory cells within the muscles of DMD patients are primarily macrophages and T cells (McDouall et al., 1990; Nitahara-Kasahara et al., 2016), although eosinophils (Cai et al., 2000), mast cells (Gorospe et al., 1994) and neutrophils (Hodgetts et al., 2006) are also present. Once in the muscle, the inflammatory cells themselves release more cytokines that orchestrate the immune response. In dystrophic muscles, cytokines contribute to a positive feedback loop of inflammation and muscle necrosis that exacerbates pathology (Heier et al., 2013; Hammers et al., 2016). Dysregulation of muscle remodelling following damage occurs due to the asynchronous occurrence of degeneration and regeneration in neighbouring fibres in dystrophic muscles, leading to inappropriate crosstalk between regions that are at different stages of the inflammatory response (Dadgar et al., 2014). Cytokines, therefore, have potential both as targets for novel therapies and as disease biomarkers.

Compared to obtaining samples by muscle biopsy, blood can be obtained by a quick, simple and relatively non-invasive technique, facilitating more frequent sampling. Quantification of proteins of which the blood concentration is altered by DMD could be used objectively to measure the body-wide response to disease or to a therapeutic intervention. The most commonly used blood biomarker is the activity of creatine kinase (CK), an enzyme that is abundant in skeletal muscles and is released into circulation following muscle damage (Hathout et al., 2015). CK activity is a highly sensitive biomarker for muscle damage and is consequently a good diagnostic marker for DMD, but can vary by orders of magnitude between and within individuals as a result of recent physical exertion (Boca et al., 2016; Coenen-Stass et al., 2016). This limits the potential utility of this biomarker in assessing DMD severity or detecting a response to treatments. Thus, the search for novel blood biomarkers for DMD has become a research focus in recent years. Many cytokines have been investigated as potential biomarkers of DMD both in muscle and blood samples. Amongst others, tumour necrosis factor-α (TNFα or TNF) and interleukin-6 (IL6) frequently show increased mRNA and protein levels within muscle tissue (Messina et al., 2011) and increased serum protein concentration in dystrophic individuals compared to healthy controls (Porreca et al., 1999; Abdel-Salam et al., 2009; Rufo et al., 2011). These cytokines are expressed by myoblasts and myotubes, as well as by inflammatory cells such as mast cells, neutrophils, macrophages and lymphocytes (Collins and Grounds, 2001; Muñoz-Cánoves et al., 2013). The levels of these cytokines are elevated within dystrophic muscle tissue as a result of increased gene expression by these cell types (Messina et al., 2011), recruitment of more inflammatory cells to the muscle (Gorospe et al., 1996) and release of stored TNFα from mast cells in response to muscle fibre damage (Radley and Grounds, 2006). In addition, large-scale serum profiling studies have highlighted the serum biomarker potential of other proteins and microRNAs associated with inflammation in human DMD patients (Hathout et al., 2015; Guiraud et al., 2017), as well as in *mdx* mouse models (Hathout et al., 2014) and the Golden Retriever muscular dystrophy (GRMD) model of DMD (Jeanson-Leh et al., 2014). Given the evidence that traditional corticosteroid anti-inflammatory treatment (Mendell et al., 1989) and treatment with more focused derivatives (Conklin et al., 2018) provide beneficial effects in DMD-affected patients, the assessment and validation of inflammatory biomarkers in animal models is especially pertinent.

Chemotactic cytokines, also referred to as chemokines, are low-molecular-mass cytokines with important roles in leukocyte extravasation and migration within tissues (Baggiolini, 2001; Mackay, 2001). They are classified into two main families, CXC and CC, depending on the configuration of N-terminal cysteines, which are adjacent in the CC family and separated by an additional amino acid in the CXC family (Baggiolini, 2001). Several studies have shown upregulated chemokine mRNA expression in the muscles of *mdx* mice (Fang et al., 2000; Porter et al., 2003) and DMD patients (Pescatori et al., 2007; De Paepe et al., 2012). Of note, muscle CCL2 expression is upregulated early in DMD patients (from 2 years of age and onwards) (Pescatori et al., 2007). Serum CCL2 concentration is higher in *mdx* mice compared to wild-type (WT) controls (Fang et al., 2000; Kranig et al., 2019) and was recently detected as being elevated in human DMD patients (Ogundele et al., 2021). To our knowledge, it has not been investigated as a serum biomarker in a large animal model of DMD.

The DE50-MD dog is a relatively newly established animal model of DMD that, unlike the *mdx* mouse and other commonly used canine models of DMD, has a point mutation within the exon 44-53 ‘hotspot’ region for dystrophin gene mutations in humans (Walmsley et al., 2010; Barthélémy et al., 2019). A natural history study has recently been completed in this model that followed dogs longitudinally for 18 months. Within the 18-month study period, DE50-MD dogs appeared to model the human DMD disease course to beyond the point of signs manifesting at 3 years of age but prior to loss of ambulation, which occurs in most DMD patients around 10-12 years of age (Duan et al., 2021). DE50-MD muscle volumes, measured every 3 months, were comparable to those of WT controls at 3 months of age, but diverged and were significantly lower than WT muscle volumes from 6 months onwards (Hornby et al., 2021).

DE50-MD muscles display classic histological features of dystrophic pathology, including widespread degeneration, regeneration, inflammation and fibrosis. The markers of degeneration and repair have been found to peak within the first year of life, with fewer foci of muscle necrosis and a reduction in the markers of regeneration from 12 months onwards, whereas fibrotic remodelling progressed more gradually (Hildyard et al., 2022). Multiple blood-borne molecules that are widely recognised as biomarkers of dystrophin deficiency in human DMD patients were also found to be significantly altered in the serum of DE50-MD dogs compared to WT controls, with peak circulating CK activity and levels of myomesin 3 (MYOM3) and the microRNA *dystromiR-206* coinciding with the period of intense degeneration and inflammation observed in muscle samples, between 3 and 9 months of age (Riddell et al., 2021). In contrast, resistance to exercise-induced muscle damage progressively declined with age (Riddell et al., 2018). Using this model, we previously revealed the potential of systemic AAV9-mediated CRISPR/Cas9 gene editing for the treatment of DMD (Amoasii et al., 2018), but longitudinal trials are required to prove the safety and efficacy of these and other methods.

The aim of this study was to quantify the expression of a panel of cytokines in the serum of DE50-MD and WT control dogs in order to establish biomarkers for future pre-clinical trials. CCL2 was further investigated as a useful biomarker that, based on the similarity of disease phenotypes, has potential for translation as a biomarker for DMD patients.

## RESULTS

### Luminex assay

#### Interleukins

A panel of 13 inflammatory proteins were assayed in multiplex by the Luminex assay in serum samples from 14 male DE50-MD dogs and 11 male WT dogs sampled longitudinally at 3-monthly intervals between 3 and 18 months of age. Of the 14 DE50-MD dogs that were followed longitudinally, six were euthanised prior to reaching the 9-month timepoint; in all instances, this was a result of reaching pre-determined humane endpoints related to dysphagia (see Material and Methods). Comparison of biomarker data from these DE50-MD dogs that were euthanised prior to 9 months of age with age-matched DE50-MD dogs that were maintained for the full 18-month study period revealed no statistical differences for any marker evaluated in this study (linear mixed model, *P*>0.05; Fig. S1; see Data Availability section for underlying raw data); thus. all data from these six animals were included in our analysis.

**Fig. 1.**
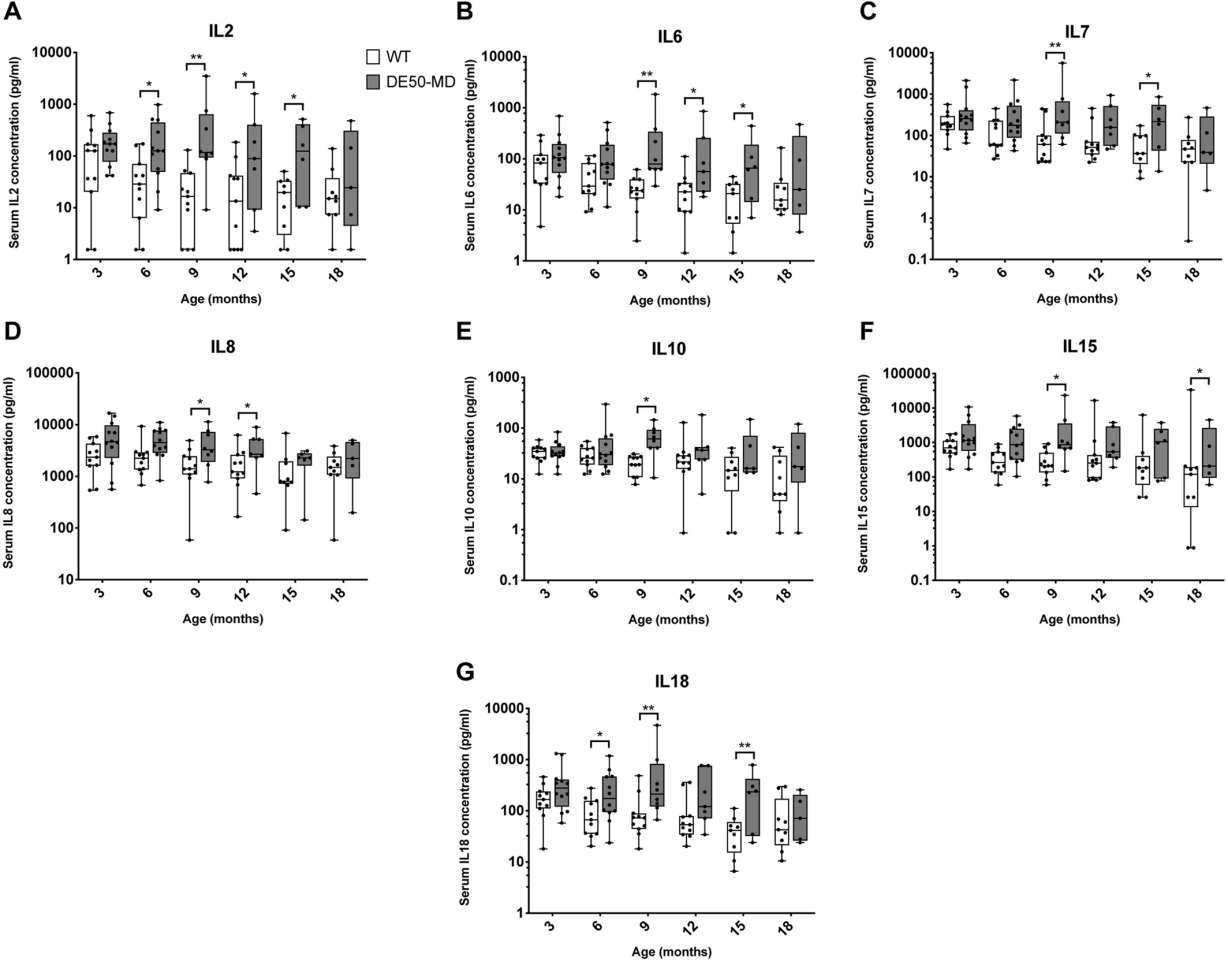
**Serum interleukin concentrations in DE50-MD and WT dogs.** Comparison of Luminex assay results for DE50-MD (grey) and WT (white) dog serum concentrations for interleukins (A) IL2, (B) IL6, (C) IL7, (D) IL8, (E) IL10, (F) IL15 and (G) IL18. Dogs were studied longitudinally between 3 and 18 months of age. Month 3: WT, *n*=11; DE50-MD, *n*=12. Month 6: WT, *n*=11; DE50-MD, *n*=12. Month 9: WT, *n*=11; DE50-MD, *n*=8. Month 12: WT, *n*=11; DE50-MD, *n*=7. Month 15: WT, *n*=9; DE50-MD, *n*=6. Month 18: WT, *n*=9; DE50-MD, *n*=5. Boxes extend from the 25th to 75th percentile, with a line within the box at the median value. Each point represents an individual DE50-MD or WT dog and whiskers show the minimum and maximum results for that age group. Asterisks indicate significance level based on linear mixed-model analysis, adjusted for repeated measures. *P<0.05; **P<0.01.

The following proteins were quantified in the Luminex assay panel: granulocyte macrophage colony stimulating factor (GM-CSF or CSF2); interferon-γ (IFNγ, encoded by *IFNG*); the interleukins IL2, IL6, IL7, IL8 (CXCL8), IL10, IL15 and IL18; interferon γ-induced protein 10 (IP-10 or CXCL10); keratinocyte chemotactic-like (KC-like, homologous to mouse CXCL1); C-C motif chemokine ligand 2 (CCL2), also known as monocyte chemoattractant protein 1 (MCP-1); and TNFα. Compared to WT dogs, DE50-MD dogs had elevated serum concentrations of all interleukins in the panel (Fig. 1). Post hoc analysis revealed that the differences between genotypes were significant at certain timepoints only, and the timepoints at which these significant differences occurred varied between interleukins. Of note, all interleukins in the panel were significantly elevated in DE50-MD dogs at 9 months of age (Fig. 1). See Table S1 for raw data from the Luminex assay, Table S2 for the means, standard deviation and range of each cytokine, and Figs S2 and S3 for longitudinal traces for individual study animals.

#### Other cytokines

**Fig. 2.**
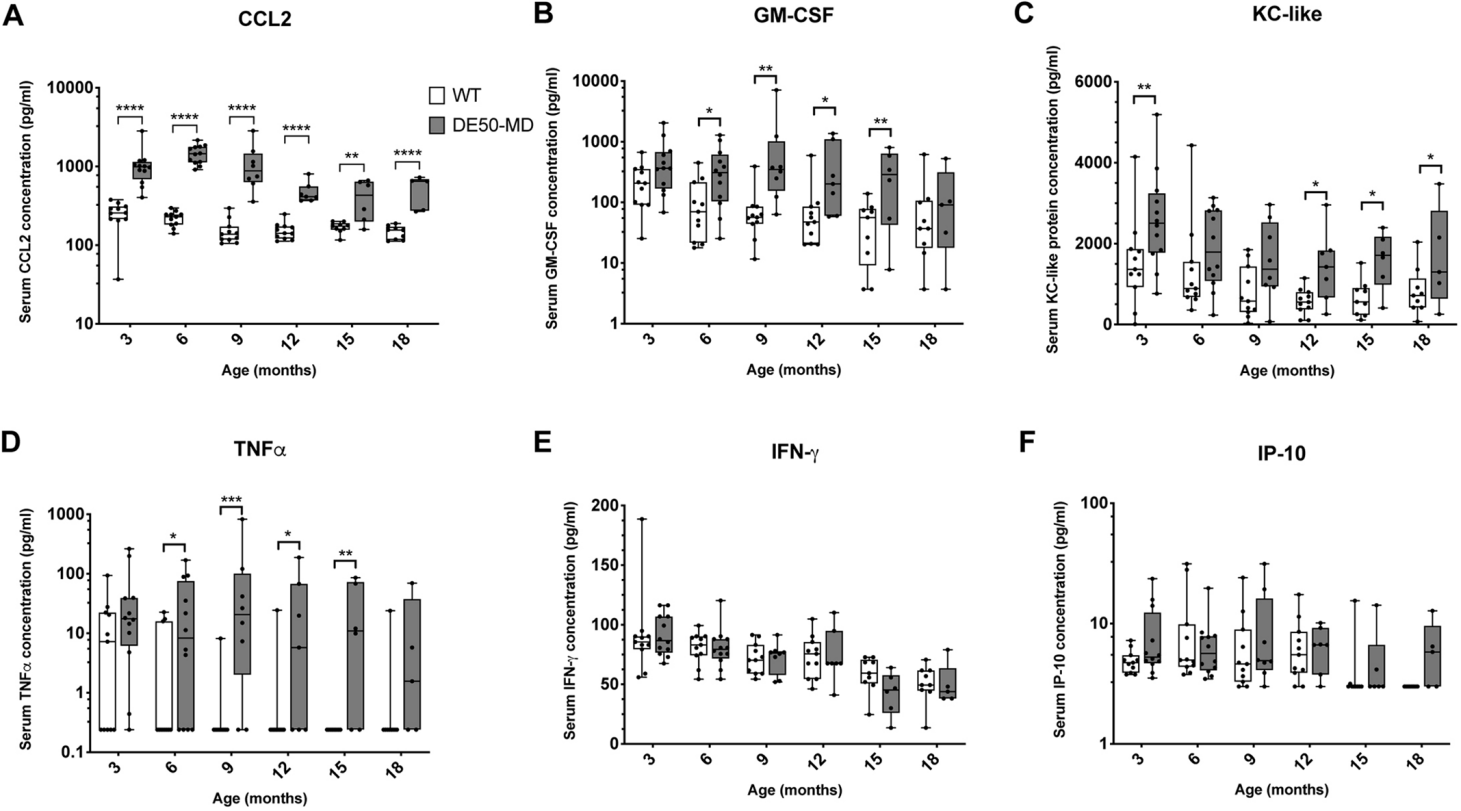
**Serum inflammatory cytokine concentrations in DE50-MD and WT dogs.** Comparison of Luminex assay results for DE50-MD (grey) and WT (white) dog serum concentrations for (A) CCL2, (B) GM-CSF, (C) KC-like protein, (D) TNFα, (E) IFNγ and (F) IP-10. Dogs were studied longitudinally between 3 and 18 months of age. Month 3: WT, *n*=11; DE50-MD, *n*=12. Month 6: WT, *n*=11; DE50-MD, *n*=12. Month 9: WT, *n*=11; DE50-MD, *n*=8. Month 12: WT, *n*=11; DE50-MD, *n*=7. Month 15: WT, *n*=9; DE50-MD, *n*=6. Month 18: WT, *n*=9; DE50-MD, *n*=5. Boxes extend from the 25th to 75th percentile, with a line within the box at the median value. Each point represents an individual dog, and whiskers show the minimum and maximum results for that age group. Results that were below the lower limit of detection of the assay are presented as the minimum detectable concentration. Concentrations varied over orders of magnitudes between and/or within groups for some of the cytokines measured; therefore, a logarithmic scale was used on the *y*-axis to better display the data for graphs A,B,D and F. Asterisks indicate significance level based on linear mixed-model analysis, adjusted for repeated measures. *P<0.05; **P<0.01; ***P<0.001; ****P<0.0001.

Several other cytokines included in the panel were elevated in the serum of DE50-MD dogs compared to WT controls: CCL2 (*P*<0.0001), GM-CSF (*P*<0.0001), KC-like (*P*=0.002) and TNFα (*P*<0.0001) were all significantly higher in DE50-MD serum (Fig. 2A-D). There was no effect of genotype on serum concentrations of IFNγ or IP-10 (*P*=0.51 and *P*=0.38, respectively) (Fig. 2E,F).

CCL2 was markedly elevated in DE50-MD serum, with the differences being the most pronounced between the ages of 3 and 12 months, a likely key age range for pre-clinical trials using this dog model (Fig. 2A). Within DE50-MD dogs, CCL2 concentration was higher at 6 months of age than all other timepoints (*P*<0.05; 6 months, 1461±391 pg/ml, indicated as mean±s.d.), with the next highest concentrations at 3 and 9 months of age (*P*<0.05; 3 months, 1051±610 pg/ml; 9 months, 1124±789 pg/ml), and lowest concentrations from 12 to 18 months of age (*P*<0.005; 12 months, 481±157 pg/ml; 15 months, 422±228 pg/ml; 18 months, 516±224 pg/ml; Fig. S4). The same age-associated pattern was not observed in WT dogs (Fig. S4). Sample size calculations showed that of the 13 proteins analysed in the Luminex panel, CCL2 best differentiated the two genotypes between 3 and 18 months of age; i.e. a sample size of only four animals per treatment group would be required to detect a 50% improvement in CCL2 concentration in DE50-MD serum compared to that in WT serum with sufficient power (power=0.8; Table 1). If this age range was restricted to 3- to 12-month-old dogs, the required sample size reduced to three animals per treatment group to detect a 50% improvement and seven animals per treatment group to detect a 25% improvement.

**Table 1.**
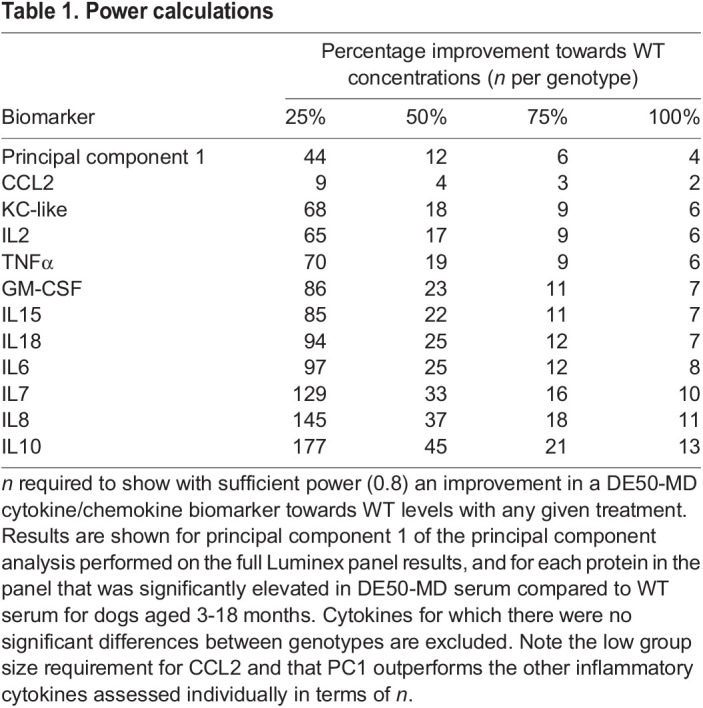
Power calculations

### Principal component analysis

**Fig. 3.**
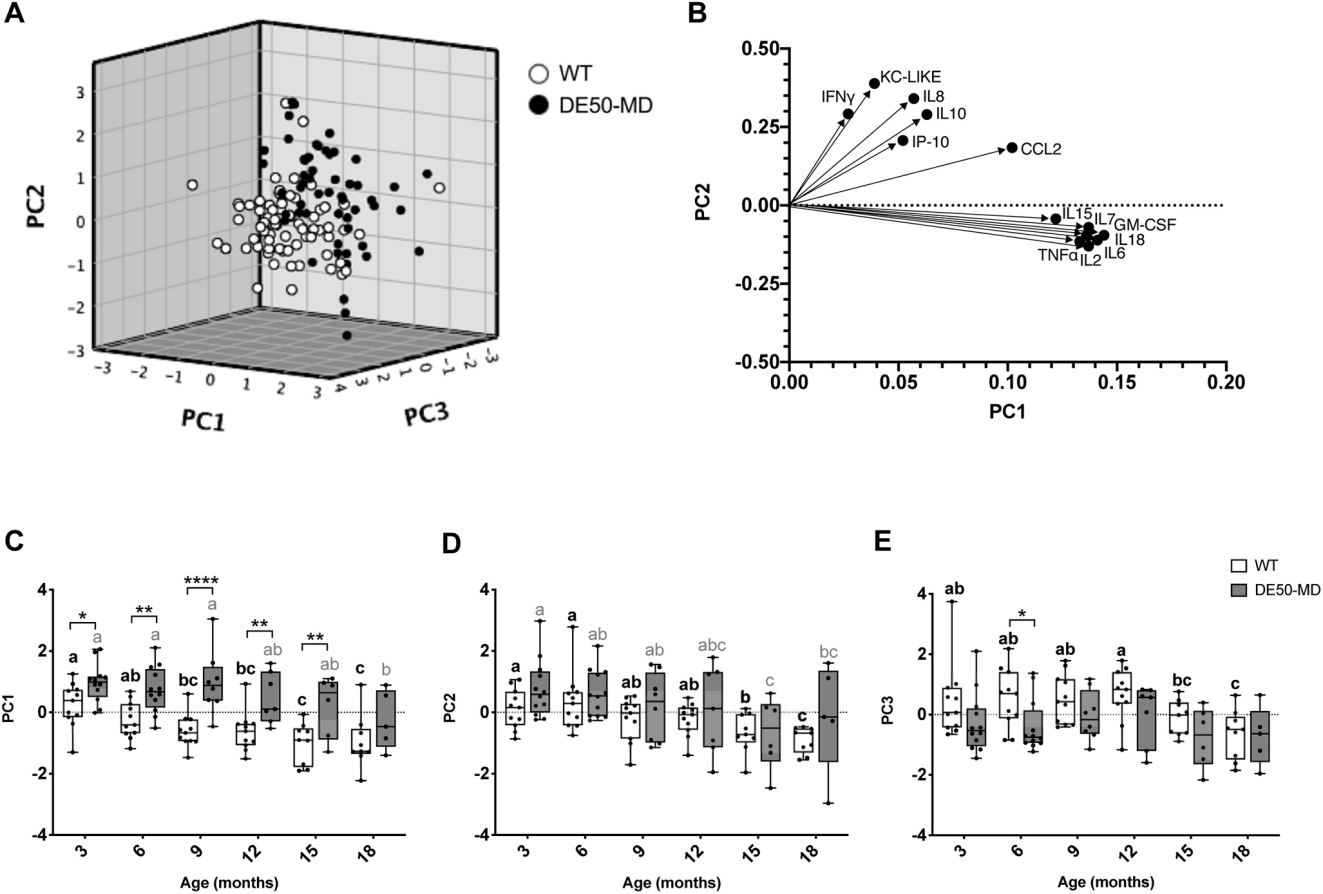
**Principal component analysis of Luminex inflammatory protein panel results.** (A) Relationship between principal components (PCs). Dogs were studied longitudinally between 3 and 18 months of age. DE50-MD (black): *n*=14 different dogs, 50 samples; WT (white): *n*=11 different dogs, 62 samples. (B) Component biplot showing relative loading of the 13 proteins to principal components 1 and 2. (C-E) Comparison of (C) PC1, (D) PC2 and (E) PC3 results for each genotype for dogs studied longitudinally between 3 and 18 months of age. Month 3: WT, *n*=11, DE50-MD, *n*=12. Month 6: WT, *n*=11; DE50-MD, *n*=12. Month 9: WT, *n*=10; DE50-MD, *n*=8. Month 12: WT, *n*=11; DE50-MD, *n*=7. Month 15: WT, *n*=9; DE50-MD, *n*=6. Month 18: WT, *n*=9; DE50-MD, *n*=5. Boxes extend from the 25th to 75th percentile, with a line within the box at the median value. Each point represents an individual dog, and whiskers show the minimum and maximum results for that age group. Asterisks denote the level of significance of a difference between genotypes based on linear mixed-model analysis, adjusted for repeated measures. *P<0.05; **P<0.01; ****P<0.0001. Letters a, b and c denote statistically significant differences (*P*<0.05) in the mean within either the DE50-MD (grey letters) or WT (black letters) genotypes; means sharing a letter are not significantly different within each genotype group.

A principal component analysis (PCA) performed using the results of all 13 proteins measured by the Luminex assay identified three principal components (PCs) that together accounted for 74.2% of the variance in the dataset: PC1, 51.7%; PC2, 12.9%; and PC3, 9.6% (Fig. 3). A three-dimensional scatter plot of PC1, PC2 and PC3 showed clustering of the two genotypes, with minimal overlap between clusters (Fig. 3A). To examine the influence of each protein on the principal components and to identify patterns between the inflammatory proteins, a component biplot showing the relative loading of the 13 proteins in the panel was produced (Fig. 3B). The component biplot showed two main clusters of proteins, with CCL2 lying between the two: the first cluster consisted of IFNγ, KC-like, IL8, IL10 and IP-10, and the second cluster consisted of IL2, IL6, IL7, IL15, IL18, GM-CSF and TNFα (Fig. 3B). Of the three components, PC1 showed the greatest disparity between genotypes between 3 and 18 months of age (Fig. 3C). PC2 showed no differences between the two genotypes but was the component most strongly influenced by age, showing a steady decline between 3 and 18 months in both WT and DE50-MD dogs (Fig. 3D). PC3 only differed significantly between genotypes at 6 months of age, but showed subtle differences in the effect of age between the two groups (Fig. 3E). Mean values for PC3 peaked at 12 months and declined thereafter in both WT and DE50-MD dogs; however, this pattern was only significant for the DE50-MD genotype. Based on these results, PC1 was investigated as a potential biomarker of dystrophin deficiency in the DE50-MD dog model; sample size calculations showed that four to six animals per treatment group would be sufficient to detect between 75% and 100% improvement in DE50-MD PC1 results towards WT levels (Table 1).

### Muscle CCL2 and CCR2 mRNA levels

**Fig. 4.**
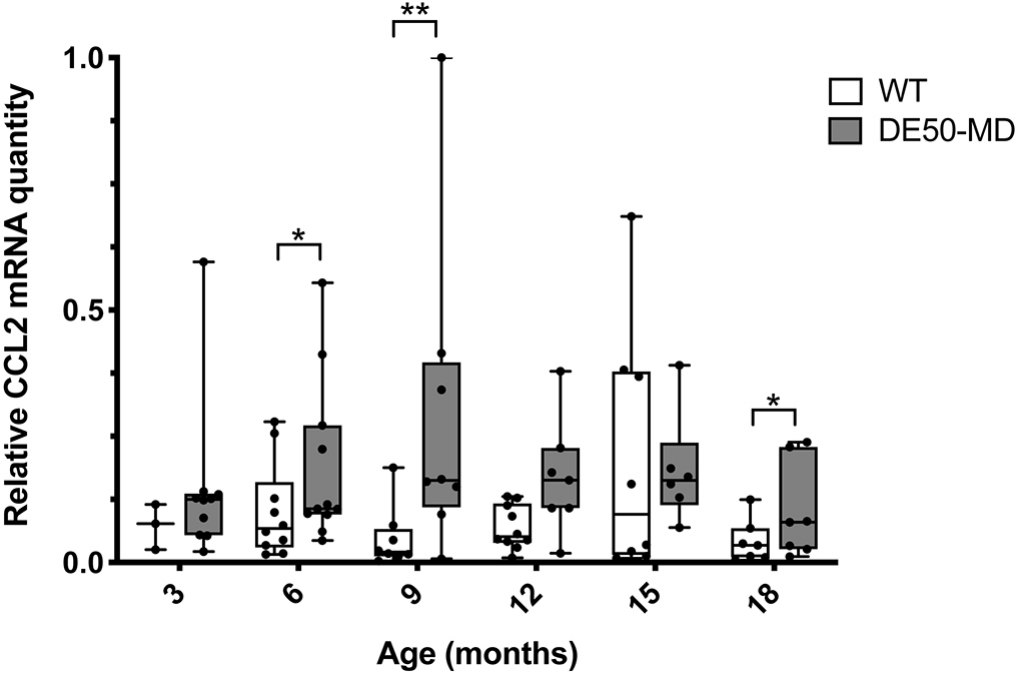
**RT-qPCR of *CCL2* mRNA in vastus lateralis muscle of DE50-MD and WT dogs.** Results were normalised to the expression of three reference genes: *RPL13A*, *HPRT1* and *SDHA*. Boxes extend from the 25th to 75th percentile, with a line within the box at the median value. Each point represents an individual DE50-MD or WT dog studied longitudinally between 3 and 18 months of age, and whiskers show the minimum and maximum results for that age group. Month 3: WT, *n*=4; DE50-MD, *n*=9. Month 6: WT, *n*=10; DE50-MD, *n*=10. Month 9: WT, *n*=10; DE50-MD, *n*=7. Month 12: WT, *n*=9; DE50-MD, *n*=6. Month 15: WT, *n*=8; DE50-MD, *n*=5. Month 18: WT, *n*=9; DE50-MD, *n*=6. Asterisks denote the level of significance of a difference between genotypes based on linear mixed-model analysis, adjusted for repeated measures. *P<0.05; **P<0.01.

Vastus lateralis muscle biopsies were obtained longitudinally at 3-monthly intervals from 3 to 18 months of age in the same cohort of animals from which serum samples were analysed by the Luminex assay (*n*=14 DE50-MD dogs; *n*=11 WT dogs). Muscle samples were not available for every dog and age group for which we had serum (see Material and Methods for full study population details), but the mRNA levels of the genes encoding CCL2 and its receptor CCR2 were quantified in all available corresponding muscle samples. The levels of *CCL2* mRNA in vastus lateralis muscle samples from DE50-MD were higher than those in WT muscles (*P*=0.002, Fig. 4). Post hoc analysis revealed that the difference was significant at 6 months (*P*=0.046), 9 months (*P*=0.002) and 18 months of age (*P*=0.032). No difference was found between genotypes in the relative levels of *CCR2* mRNA (*P*=0.84; Fig. S5).

**Fig. 5.**
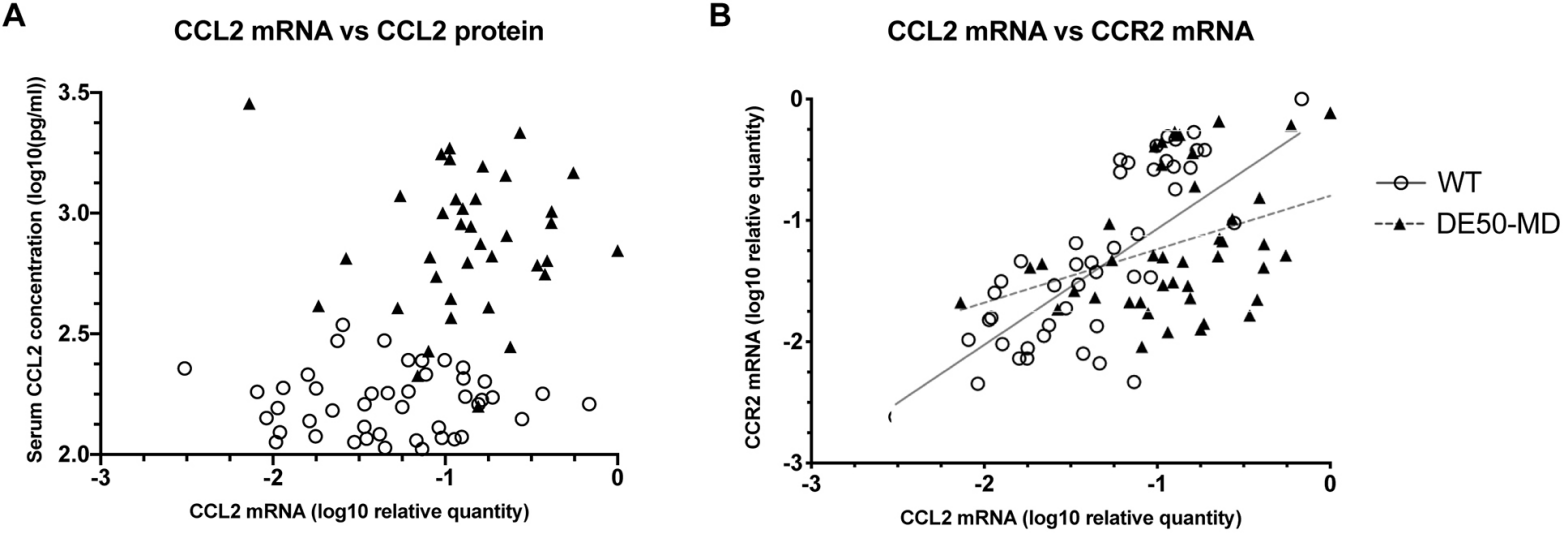
**Relationship between serum CCL2 protein and vastus lateralis muscle *CCL2* mRNA.** Graph relationships between the relative levels of *CCL2* mRNA in the vastus lateralis muscle and serum CCL2 protein concentration (A), and between the relative *CCL2* and *CCR2* mRNA levels in the vastus lateralis muscle (B) of DE50-MD (black triangles) and WT (open circles). No relationship was found between serum CCL2 protein concentration and vastus lateralis muscle *CCL2* mRNA levels within genotypes (DE50-MD: *P*=0.59, *n*=36 samples from a total of 13 dogs; WT: *P*=0.95, *n*=45 samples from a total of 11 dogs). A positive association between *CCL2* and *CCR2* mRNA levels in the vastus lateralis muscle was found for both WT (*P*<0.0001, *n*=49 samples from a total of 11 dogs) and DE50-MD (*P*=0.001, *n*=42 samples from a total of 13 dogs) subjects, based on linear mixed-model analysis. Repeated measures were accounted for in the statistical analyses.

There was no significant relationship between the relative levels of muscle *CCL2* mRNA and serum CCL2 concentration within either genotype (DE50-MD, *P*=0.87; WT, *P*=0.93; Fig. 5A). There was a significant positive relationship between the relative mRNA levels of *CCL2* and *CCR2* in vastus lateralis muscle samples [WT, R^2^=0.75, *P*<0.0001, slope gradient estimate=1.10±0.15 (±s.e.); DE50-MD: R^2^=0.62, *P*=0.0004, slope estimate=0.66±0.17) (Fig. 5B).

### Correlation of serum CCL2 concentration with other biomarkers of disease

Previous work analysing samples from the same cohort of dogs as the present study showed that plasma CK activity (Riddell et al., 2021) and the acid phosphatase-stained fraction of the vastus lateralis muscle (Hildyard et al., 2022) were markedly higher in DE50-MD dogs compared to WT dogs. Acid phosphatase is a lysosomal enzyme abundant in macrophages and neutrophils, but also found in degenerating/regenerating muscle, and thus is a frequently used marker of inflammation in muscle tissue (Dubowitz et al., 2021). We compared the CK activity and acid phosphatase results from earlier studies (Riddell et al., 2021, Hildyard et al., 2022, respectively) with the serum CCL2 concentration determined in the present study. Both CCL2 concentration and CK activity were quantified in blood taken from the same dog at the same timepoint. Similarly, serum CCL2 concentration was compared to vastus lateralis muscle acid phosphatase staining from tissues obtained from the same dog at the same timepoint.

#### Blood-borne CCL2 concentration and CK activity

**Fig. 6.**
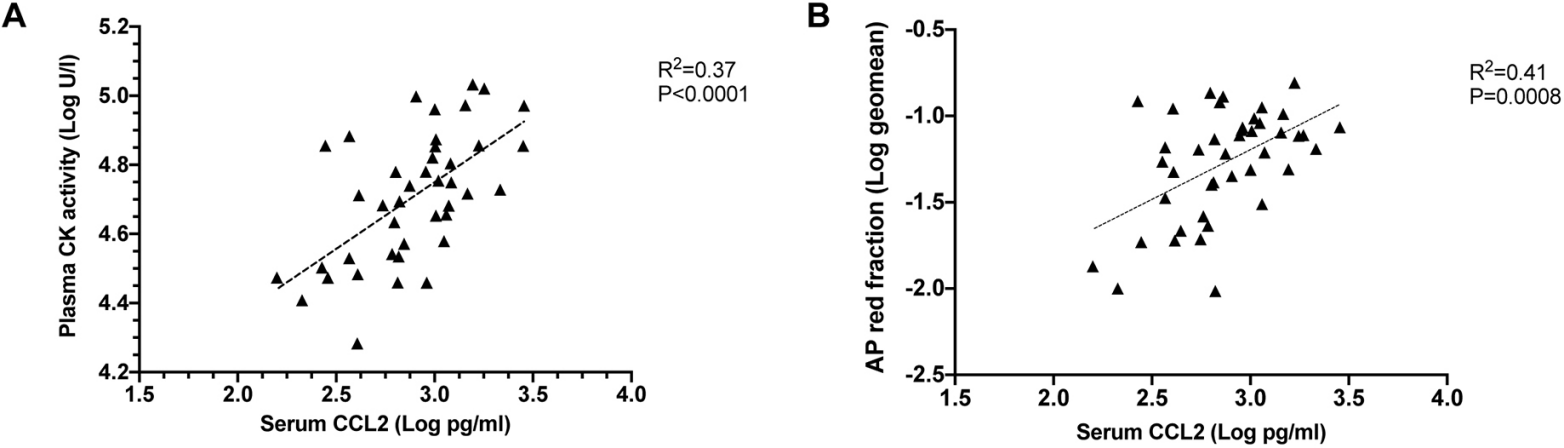
**Relationship between serum CCL2 protein concentration and other biomarkers of disease in DE50-MD dogs.** (A) Graph relationship between serum CCL2 concentration and plasma CK activity. A positive relationship was found between the two variables (R^2^=0.37, *P*<0.0001). *n*=41 samples, from a total of 14 DE50-MD dogs studied longitudinally between 3 and 18 months of age (month 3, *n*=11; month 6, *n*=9; month 9, *n*=7; month 12, *n*=5; month 15, *n*=5; month 18, *n*=4). (B) Graph relationship between serum CCL2 concentration and the geometric mean (geomean) of acid phosphatase-stained fraction of vastus lateralis muscle biopsy samples. A positive relationship was found between the two variables in DE50-MD dogs (R^2^=0.41, *P*=0.0008). *n*=42 samples, from a total of 14 DE50-MD dogs studied longitudinally between 3 and 18 months of age (month 3, *n*=7; month 6, *n*=10; month 9, *n*=8; month 12, *n*=7; month 15, *n*=5; month 18, *n*=5). Linear regressions were calculated based on linear mixed-model analysis, accounting for repeated measures.

For DE50-MD dogs, a positive relationship was found between serum CCL2 concentration (pg/ml) and plasma CK activity (U/l) (R^2^=0.37, *P*<0.0001, slope gradient estimate=0.39±0.09; *n*=41 samples from a total of 14 dogs; Fig. 6A). No correlation was found in WT samples (*P*=0.70; *n*=46 samples from a total of 11 dogs; Fig. S6A). See Data Availability section for underlying data.

#### Blood-borne CCL2 concentration and muscle acid phosphatase staining

A positive relationship was found between the serum CCL2 concentration and acid phosphatase-stained fraction of vastus lateralis muscle biopsy sections in DE50-MD dogs (R^2^=0.41, *P*=0.0008, slope gradient estimate=0.58±0.17; *n*=42 samples from a total of 14 dogs; Fig. 6B). No correlation was found between the two variables within WT dogs (*P*=0.66, *n*=55 samples from a total of 11 dogs; Fig. S6B). See Data Availability section for underlying data.

### CCL2 immunohistochemistry

**Fig. 7.**
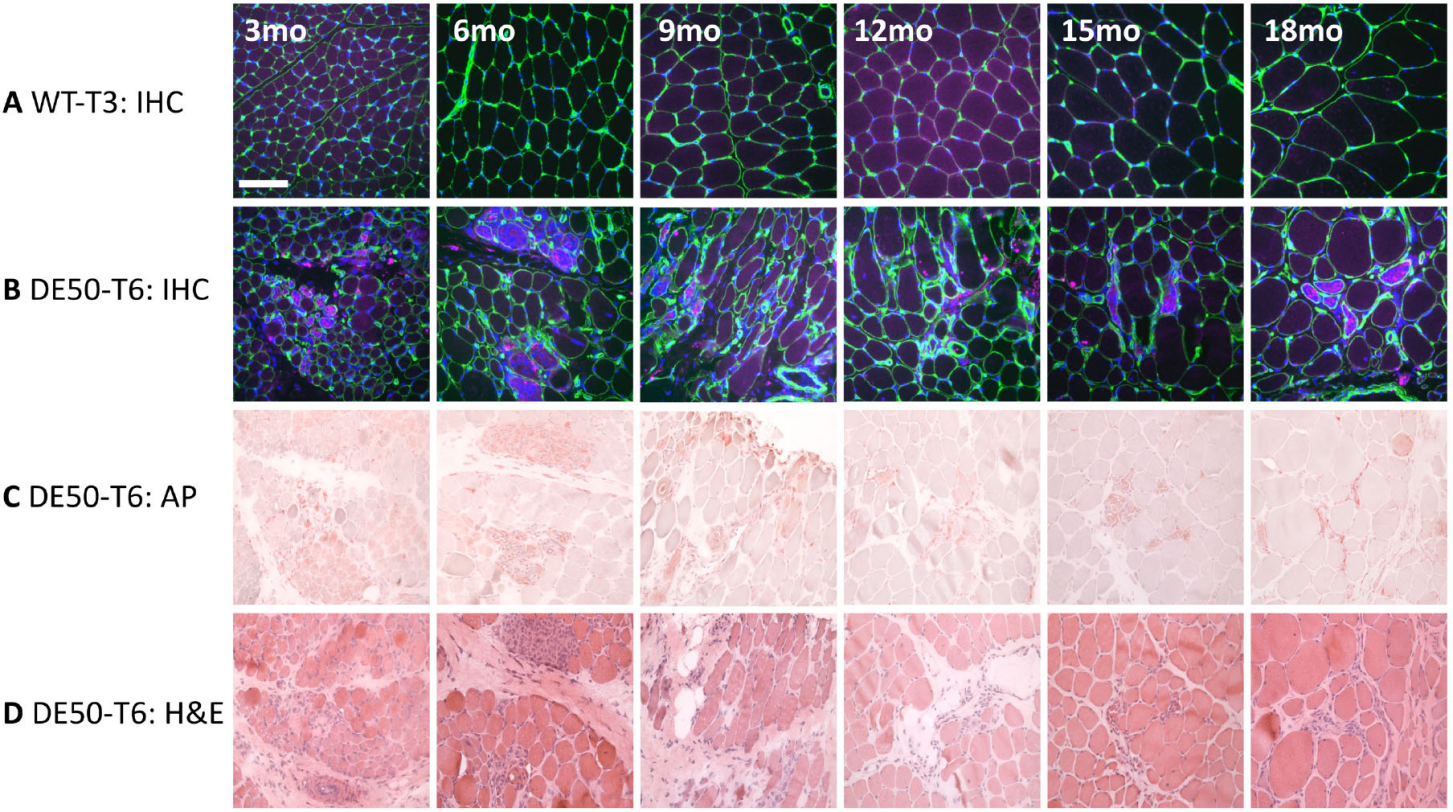
**Histology of the vastus lateralis muscle from WT and DE50-MD littermates from 3 to 18 months of age.** (A,B) Immunohistochemistry (IHC) of vastus lateralis muscle from (A) a WT dog (ID WT-T3) and (B) a DE50-MD dog (ID DE50-T6). Each section was labelled with antibodies against perlecan (green) and CCL2 (magenta), and nuclei were labelled with Hoechst 33342 (blue). (C,D) For the DE50-MD dog DE50-T6, serial sections for each timepoint were stained for (C) acid phosphatase (AP) and with (D) H&E. Scale bar: 100 µm.

Vastus lateralis muscle biopsies from a WT dog and a DE50-MD littermate (dog IDs WT-T3 and DE50-T6, respectively) sampled longitudinally at 3-monthly intervals from 3 to 18 months of age were analysed. To identify regions of inflammation, serial sections underwent Haematoxylin and Eosin (H&E) staining, acid phosphatase staining (lysosomal marker) and CCL2 immunohistochemistry. Muscle sections showed groups of muscle fibres that were positively labelled for CCL2 in DE50-MD samples but not in WT samples (Fig. 7A,B; no primary control, Fig. S7). The CCL2-positive areas showed signs of degeneration and regeneration, including internalised nuclei and fibre-size variation (Fig. 7B). Scattered CCL2-positive mononuclear cells were also seen in higher numbers in the DE50-MD sections compared to WT sections (Fig. 7B). The areas of muscle that were positive for CCL2 were also positive for acid phosphatase (Fig. 7C), and often showed dense cell infiltration by H&E staining (Fig. 7D). Acid phosphatase staining was not observed in the WT muscle samples (Fig. S8).

**Fig. 8.**
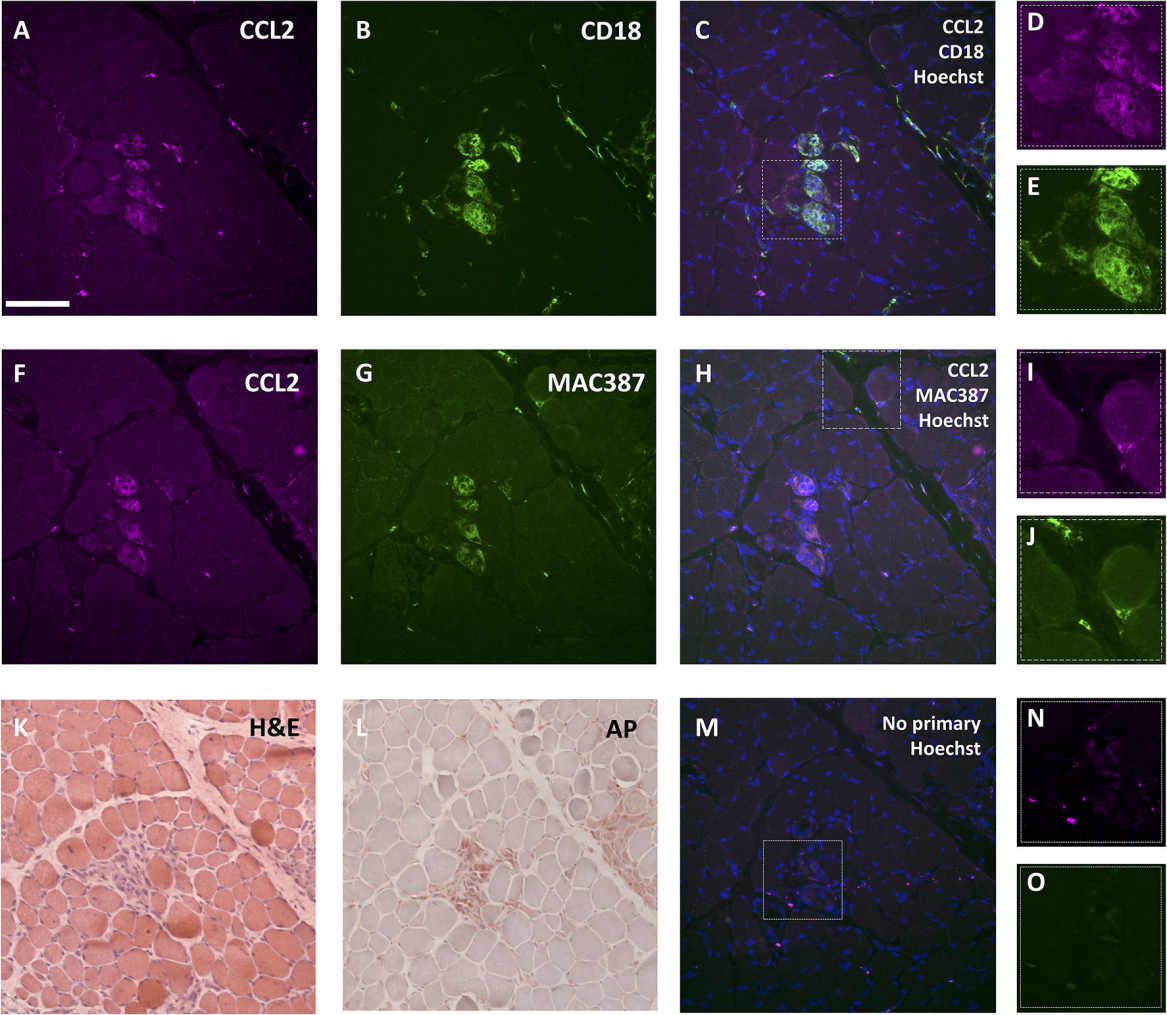
**Histology of the vastus lateralis muscle from a 6-month-old DE50-MD dog.** (A-E) Immunohistochemistry of a section of vastus lateralis muscle showing (A) CCL2 (magenta), (B) CD18 (green) and (C) a merged image of CCL2, CD18 and Hoechst 33342 (blue), with magnified views of the boxed region in C shown for (D) CCL2 and (E) CD18 staining. (F-J) Immunohistochemistry of a serial section showing (F) CCL2 (magenta), (G) MAC387^+^ regions (green) and (H) a merged image of CCL2, MAC387 and Hoechst 33342 (blue), with magnified views of the boxed region in H shown for (I) CCL2 and (J) CD18 staining. (K) A serial section with H&E staining showing cell infiltrate within and between muscle fibres, fibres with internalised nuclei and hypercontracted fibres (darker magenta). (L) Serial section with acid phosphatase (AP) staining showing positive red staining at areas of cell infiltrate and within some muscle fibres. (M-O) Serial section of immunohistochemistry with no primary antibody as a control with magnified views of the region of inflammation (dashed box) shown for the (N) magenta (CCL2) and (O) green (CD18/MAC387) channels. Scale bar: 100 µm.

To further define the inflammatory milieu of the sample, immunohistochemistry was performed to locate the pan-leukocyte marker CD18 (ITGB2) and the macrophage, monocyte and neutrophil marker antibody MAC387, alongside the chemokine CCL2. CCL2 typically colocalised with both CD18 and MAC387 (Fig. 8A-J). These three markers were present within muscle fibres that were infiltrated with mononuclear cells and likely undergoing necrosis, as shown by H&E and acid phosphatase staining of serial sections (Fig. 8K,L). The specificity of secondary antibody binding was confirmed by inclusion of a no-primary-antibody control stain of a serial section of muscle (Fig. 8M-O). CCL2^+^/CD18^+^ and CCL2^+^/MAC387^+^ single cells were also present within the perimysium and at the periphery of muscle fibres within fascicles (Fig. 8A-J). Serial sections showed that cells and areas that were positive for CD18 were often also positive for MAC387 (Fig. 8B,G). However, not all CD18^+^ and MAC387^+^ cells expressed detectable levels of CCL2 (Fig. 8D,I), indicating that a mixed population of inflammatory cells was present. In addition, certain muscle fibres appeared to express CCL2 in the absence of inflammatory cell infiltrate (Fig. 8D), indicating that the muscle fibre itself is a source of CCL2.

## DISCUSSION

Blood sample analysis offers a minimally invasive way to monitor disease or a response to treatment in both DMD patients and pre-clinical animal models. Moreover, blood biomarkers assess whole-body responses rather than regional changes that are examined in, for example, histological evaluation of muscles. As chronic inflammation is a feature of dystrophic muscles, and given the renewed interest in novel anti-inflammatory therapeutics (Dort et al., 2021; Raimondo and Mooney, 2021; Xu et al., 2021), here, we examined a series of serum cytokines in the blood of dystrophic DE50-MD dogs in comparison with WT littermates. Concentrations of CCL2, GM-CSF, KC-like protein, TNFα and interleukins IL2, IL6, IL7, IL8, IL10, IL15 and IL18 were all significantly higher in DE50-MD serum compared to WT serum. This supports the hypothesis that, like DMD patients, the DE50-MD model undergoes inflammation associated with the dystrophic phenotype (Schmalbruch, 1984). CCL2 in particular shows promise as a useful biomarker for therapeutic trials in this colony; furthermore, it might be a useful biomarker in DMD patient trials.

For the majority of proteins in the panel, the mean serum concentration peaked at 9 months of age in DE50-MD dogs. Several other blood-borne biomarkers of the DE50-MD phenotype, including CK activity and myomesin 3 concentration, also show an early peak between 6 and 9 months of age and a subsequent decline in concentration through to 18 months of age (Riddell et al., 2021). This age-associated pattern mimics that of multiple serum biomarkers in DMD patients: the concentration of circulating biomarkers often peaks relatively shortly after the onset of clinical signs and decreases later in disease progression, likely as a result of reduced muscle mass and reduced patient activity (Saito et al., 2000; Hathout et al., 2015; Rouillon et al., 2015). In both DE50-MD dogs and their WT littermates, overall muscle volume increases until dogs reach 9 or 12 months of age and plateaus thereafter (Hornby et al., 2021); however, it is likely that not all of that muscle volume is functional muscle tissue in DE50-MD dogs. Indeed, DE50-MD muscles show changes on magnetic resonance imaging, indicative of an increasing proportion of normal muscle tissue being replaced with fibrosis and/or other infiltrates (Hornby et al., 2021). Therefore, affected dogs likely have less functional muscle tissue that can be damaged and become inflamed, as they age.

Another explanation for the peak in inflammatory biomarkers could be that, as seen in *mdx* mice and GRMD dogs, there might be a critical period of muscle degeneration around 9 months of age in the DE50-MD model, following which the disease stabilises. In the *mdx* mouse, an intense period of muscle necrosis occurs from 3 to 6 weeks of age, after which robust regeneration causes an improvement in phenotype and stabilisation up to 13 months of age (Pastoret and Sebille, 1995). Similarly, the GRMD model shows a period of destabilisation with marked myofibre necrosis and muscle atrophy between 3 and 6 months of age (Valentine et al., 1988; Shimatsu et al., 2005; Barthélémy et al., 2014), with the disease subsequently progressing more slowly in many cases (Kornegay et al., 2012; Kornegay and Childers, 2016). Compared to DMD patients, animal models of the disease have a much shorter growth phase: the *mdx* mouse model reaches adulthood at approximately 6 weeks of age, and it is during this intense growth period that peak muscle necrosis occurs (Duddy et al., 2015). As the decline in serum cytokine concentrations from 12 months onwards also coincides with the age at which maximum muscle volumes are reached in the DE50-MD dog, it is possible that cessation of muscle growth might contribute to a reduction in the severity of dystrophic pathology in this model. This current study forms part of a larger natural history study of the DE50-MD dog model that aims to provide a comprehensive characterisation of the disease phenotype. Data from this study and preliminary results from other biomarker assays suggest that DE50-MD dogs initially perform similarly to age-matched WT littermates for many biomarkers; however, between 3 and 12 months, biomarker results diverge markedly between the two genotypes, followed by a more gradual progression thereafter (Hornby et al., 2021; Riddell et al., 2021; Hildyard et al., 2022). Histological analysis of DE50-MD muscle strongly supports the notion that there is a peak degeneration and regeneration phase between 3 to 9 months of age, followed by a reduction in markers of inflammation, necrosis and regeneration from 12 months onwards (Hildyard et al., 2022). A subset of the DE50-MD dogs included in this study were euthanised due to reaching pre-determined humane endpoints prior to the planned 18-month end-date. In all cases, the criterion reached was related to dysphagia. We had considered that these animals might display greater severity in other aspects of their phenotype; however, beyond this specific sign, the animals appeared comparable to non-dysphagic dystrophic animals and no statistically significant differences were found between these two groups of animals in any of the proteins or mRNAs analysed in this study.

Although mean serum concentration of all 13 inflammatory proteins in the panel was higher in DE50-MD dogs than in WT dogs at 3 months of age, the difference between genotypes only reached statistical significance for CCL2 and KC-like protein, suggesting that these two proteins could be useful early biomarkers of the DE50-MD phenotype. Both proteins are chemokines produced by both a range of leukocytes as well as skeletal muscle tissue itself, and recruit leukocytes to sites of inflammation (Hua et al., 2005; Yahiaoui et al., 2008). A link between serum concentrations of CCL2 and KC-like has previously been demonstrated in a study comparing serum inflammatory profiles of dogs affected by the protozoan parasite *Babesia canis*; these two proteins were the only analytes in a Luminex assay panel consisting of IL2, IL7, IL8, IL10, IL15, IL18, GM-CSF, KC-like and CCL2 that could significantly differentiate uncomplicated and complicated cases, and, thus, had the most prognostic value as biomarkers of disease outcome (Galán et al., 2018).

A PCA was performed in an attempt to summarise the serum cytokine profile of the DE50-MD dog. The PCA showed clustering of the cytokine panel data by genotype, and identified a component that accounted for 50% of the total variability in the dataset and significantly differed between the two genotypes from 6 to 15 months of age. This principal component showed better discrimination between the two genotypes than the majority of proteins in the panel when looked at individually, and therefore could be used in future studies as a biomarker of body-wide inflammation in the DE50-MD dog. Power calculations support the use of PC1 as a method of distinguishing DE50-MD from WT animals, but suggest that it might not be a sensitive enough marker to detect modest phenotypic improvements in pre-clinical trials.

Of the 13 proteins in the inflammatory panel, CCL2 best differentiated the two genotypes across the 3- to 18-month study period. CCL2 is a chemokine expressed by injured skeletal muscle, macrophages, neutrophils and a variety of other cell types (Yahiaoui et al., 2008; Capucetti et al., 2020). Its primary function is the recruitment of macrophages to sites of muscle injury; however, it also contributes to muscle regeneration and recovery of function following injury by acting directly on myoblasts via the CCR2 receptor (Warren et al., 2004; Yahiaoui et al., 2008; Lu et al., 2011). Previous work has shown increased levels of serum and muscle CCL2 protein in *mdx* mice (Kranig et al., 2019). Recently, increased levels of serum CCL2 were reported in a group of DMD patients (Ogundele et al., 2021); however, its physiological significance and clinical utility as a DMD biomarker in humans is yet to be demonstrated. We found a positive linear relationship between serum CCL2 concentration and CK activity, a commonly used DMD biomarker, in DE50-MD dogs. However, the correlation was relatively weak, likely reflecting different mechanisms and timescale of release into circulation following muscle damage. As CK release can result from even minor disruptions to sarcolemmal integrity (Costa et al., 2009), perhaps a higher threshold of muscle damage, with subsequent downstream and sustained inflammatory responses, might result in increased levels of circulating CCL2.

Compared to the other biomarkers quantified in this study, fewer dogs would be required per treatment group of a pre-clinical trial in order to identify a reduction in the concentrations of CCL2 towards WT levels with sufficient power. Due to the variation within the DE50-MD population, the majority of inflammatory proteins would have to reach a 75-100% resolution in serum concentrations in order to show a significant effect of treatment with a feasible sample size of dogs. Although improvements of this magnitude are desirable, it is important that we identify biomarkers that are able to detect more subtle improvements in response to therapy, as mild to moderate functional improvements might have profound beneficial consequences for DMD patients. We found little to no overlap in the range of serum CCL2 concentration for DE50-MD versus WT groups between 3 and 18 months of age; hence, sample size calculations suggest that as few as nine dogs would be required to detect a 25% improvement in serum CCL2 levels in DE50-MD towards WT levels, and that this number could be reduced further if the age range is restricted to 3-12 months of age.

Increased concentration of *CCL2* mRNA in skeletal muscles has been demonstrated in both *mdx* mice and DMD patients (Porter et al., 2003; Pescatori et al., 2007). Muscle biopsy analysis confirmed that DE50-MD dogs also had a higher quantity of *CCL2* mRNA compared to that in WT, but mRNA expression levels did not correlate with serum CCL2 concentration within genotypes. This lack of correlation could indicate that muscle CCL2 and serum CCL2 are not physiologically linked. Alternatively, as DMD does not affect all muscles homogenously (Straub et al., 1997; Chrzanowski et al., 2017), it could indicate that serum CCL2 is not an accurate reflection of CCL2 concentrations within the specific muscle that was sampled in this study (vastus lateralis muscle). Or, given the focal nature of inflammation seen histologically (acid phosphatase staining, CD18 and CCL2), the discrepancy could indicate that serum CCL2 does not reflect *CCL2* mRNA levels in the small sample of vastus lateralis muscle (0.5 cm^3^) collected at each timepoint. Muscle biopsy is by necessity only a sampling of broader muscle pathology. As pathology is not homogenously distributed throughout muscle, a given biopsy from a dystrophic individual might sample a region dominated by inflammation, fat, fibrotic scarring or a rare region of relatively healthy muscle, giving different pictures of ongoing pathology as a consequence. In contrast, serum is a homogenous solution offering assessment of body-wide pathology and is, therefore, more likely to reflect generalised pathology at the time of collection than a muscle biopsy collected from a single muscle.

Histological analysis performed on serial sections of DE50-MD muscle showed that the CCL2 protein was found primarily in areas of muscles with signs of recent or ongoing degeneration. Regions of inflammation were identified by H&E and acid phosphatase (a lysosomal marker) staining. Acid phosphatase staining was found both within and at the periphery of muscle fibres, frequently colocalising with foci of mononuclear cell infiltrates, in line with its known expression in lysosomes of degenerating/regenerating muscles, macrophages and neutrophils (Dubowitz et al., 2021). CCL2 was similarly found within mononuclear cells, in the endomysial matrix and within fibres themselves, suggesting that muscle fibres might be a source of CCL2. Staining for the pan-leukocyte marker CD18 confirmed the presence of leukocyte lineages within areas of degenerating muscle. CD18 staining frequently colocalised with CCL2. Some muscle fibres, however, were positive for CCL2 in the absence of CD18, implying that muscle fibres do indeed represent an additional source of this chemokine. CD18- and CCL2-positive muscle fibres and single cells also frequently bound MAC387, an antibody that recognises the calcium-binding protein MRP14 (S100A9) (Goebeler et al., 1994) that is present on monocytes, some macrophages and neutrophils (Shimizu et al., 2011). However, some strongly MAC387-positive cells showed no detectable CCL2 staining, likely reflecting the range of different cell types that express the MAC387 target protein and the presence of macrophages in different forms or phases of activation. In addition to macrophages, neutrophils might also contribute to CCL2 expression and secretion within DE50-MD muscles; both dog and human leucocyte populations consist of a high percentage of neutrophils (50-70% of all leukocytes) compared to mice (10-25%) (Mestas and Hughes, 2004; Papasouliotis et al., 2006). Furthermore, a study in the GRMD model of DMD showed that neutrophil content was 35-fold higher than in WT control dog muscle (Terrill et al., 2016), implying a significant contribution of neutrophils to dystrophic pathology in dogs. Overall, these results align with the known functions of CCL2 and mimic immunohistochemistry results from *mdx* mouse muscles, which show the CCL2 protein both within and surrounding damaged muscle fibres and its colocalisation with regions of mononuclear cell infiltrates (Porter et al., 2003). A positive correlation was found between DE50-MD dog serum CCL2 and the extent of acid phosphatase staining in age-matched vastus lateralis muscle sections, further supporting a link between circulating CCL2 and the inflammatory histological phenotype.

Elevated serum CCL2 not only provides us with a non-invasive serum biomarker of dystrophin deficiency in the DE50-MD model, but also a potential therapeutic target. CCL2-induced recruitment of inflammatory cells to damaged muscles helps orchestrate repair and regeneration (Lu et al., 2011). However, the chronic presence of leukocytes as a consequence of the continuous bouts of degeneration in dystrophic muscles is associated with exacerbation of myonecrosis, impaired regeneration and accumulation of fibrosis (Rando, 2001; Cappellari et al., 2020). Inhibition of CCL2 signalling has the potential to reduce disease severity by reducing leukocyte infiltration (Liang et al., 2018). Antagonism of the CCL2 receptor, CCR2, ameliorates histopathological features and increases muscle strength in *mdx* mice (Liang et al., 2018). However, evidence that CCR2 inhibition provides sustained improvement of pathology is currently lacking, potentially due to the inhibition of the direct role of CCL2 in muscle repair (Zhao et al., 2017). Thus, addressing the balance of chemokine ligands and receptors, rather than full inhibition of specific proteins, is a strategy that could be explored in the DE50-MD model.

In conclusion, this work provides evidence that elevated levels of serum inflammatory proteins are a feature of the dystrophic phenotype of the DE50-MD model of DMD. We propose that CCL2 in particular could represent a valuable serum biomarker in the DE50-MD model. Future pre-clinical studies will determine whether an improvement in muscle pathology and function in treated animals is associated with a decrease in circulating CCL2.

## MATERIALS AND METHODS

### Animal husbandry

Dogs used in this study were from the DE50-MD colony, housed in a dedicated canine facility at the Royal Veterinary College, London. Dogs were group-housed indoors in large pens with daily access to outdoor paddocks (12 h/12 h light/dark cycle, 15-24°C): conditions that exceed the minimum stipulations of the Animal (Scientific Procedures) Act 1986 and according to local Animal Welfare Ethical Review Body approval. Carrier female Beagle-cross dogs, derived from an original founder Bichon-Frise cross Cavalier King Charles Spaniel female carrier, were mated with male Beagles (RCC strain, Marshall BioResources) to produce dogs for this study. Pregnant females (single housed) whelped naturally and puppies were kept with the mother in a large pen with a heat lamp (∼28°C) to allow nursing. Puppies were reared by their mother until approximately 4 weeks, after which they were transitioned to puppy food suitable to requirements. Puppies under 6 months of age were fed at least three times daily, initially with Royal Canin Puppy ProTech Colostrum with milk mixed with Burns Puppy Original Chicken and Rice, and transitioning to Burns Puppy Original Chicken and Rice mixed with Royal Canin Gastro-Intestinal Puppy Food (2:1). From 6 months onwards, dogs received two feeds daily of Burns Puppy Original Chicken and Rice mixed with Royal Canin Gastro-Intestinal Puppy Food (2:1) and *ad lib* water. Dogs received daily human interaction and underwent a comprehensive socialisation programme. Dogs not required for studies were rehomed.

### ARRIVE statement

Animal Research: Reporting of *In Vivo* Experiments (ARRIVE) guidelines were followed for the design and conduct of the study. All experimental procedures involving animals in this study were conducted according to UK legislation, within a project licence (P9A1D1D6E, granted 11 June 2019) assigned under the Animal (Scientific Procedures) Act 1986 and approved by the Royal Veterinary College Animal Welfare Ethical Review Body. All efforts were made to minimise any animal suffering throughout the study. Dogs were observed daily by animal technician staff and any concerns regarding animal health/welfare were reported to and assessed by the Study Director, the Named Veterinary Surgeon and the Named Animal Care and Welfare Officer.

### Study population

**Fig. 9.**
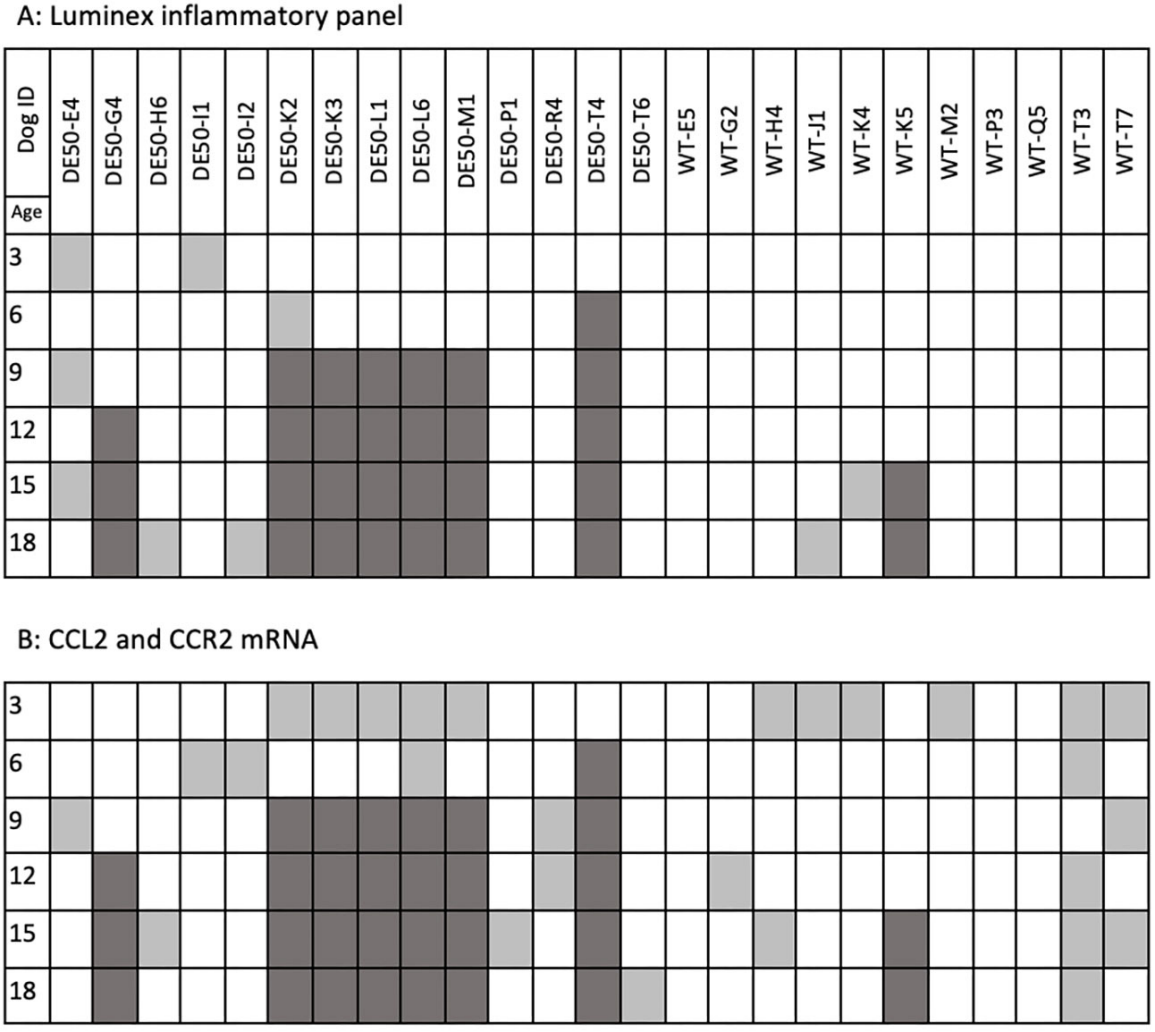
**Samples tested for each study dog.** (A) Serum samples tested by Luminex assay and (B) vastus lateralis muscle samples assayed by RT-qPCR for quantification of *CCL2* and *CCR2* mRNA. Age is displayed in months. Dog ID shows the study name of each individual animal included in this study: dog IDs with the prefix ‘DE50-‘ are DE50-MD dogs, dogs with the prefix ‘WT’ are wild-type dogs. White boxes show samples that were tested. Grey boxes indicate untested samples: dark grey boxes indicate samples that were unavailable due to animal euthanasia prior to this timepoint, whereas light grey boxes indicate samples unavailable for other reasons.

In total, data from 14 male DE50-MD dogs and 11 male WT dogs aged between 3 and 18 months are included in this study (Fig. 9). The sample size for the study was determined in order to generate an accurate estimation of the variance for each timepoint and genotype to enable future sample size calculations, as reported in this paper. Samples (serum/muscle tissue) were not available for all dogs at all timepoints in some cases due to euthanasia prior to the end of the 18-month study period (Fig. 9). Of the 14 DE50-MD dogs enrolled in the study, six were euthanised prior to reaching the 9-month timepoint as a result of reaching pre-determined humane endpoints related to dysphagia (Fig. 9). A further two animals (one healthy, WT-K5; one DE50-MD, DE50-G4) were euthanised prior to study completion for unrelated reasons (Fig. 9). Euthanasia was performed using an overdose of sodium pentobarbital (250 mg/kg, Dolethal, Covetrus) administered intravenously via preplaced catheter. Missing datapoints did occur for reasons other than euthanasia for certain timepoints, primarily owing to exhaustion of the finite supply of tissue sample collected at a specific timepoint, unless otherwise stated.

### Genotyping

Genotyping was performed on cheek-swab-derived DNA by PCR and Sanger sequencing as previously described (Amoasii et al., 2018). Briefly, a bristled cheek swab was used to collect cells from the inside of the dog's cheek. Genomic DNA was then extracted from the cells and the region of the DMD gene containing the DE50-MD mutation was amplified by PCR. PCR products underwent Sanger sequencing to confirm genotype (GATC Biotech/Eurofins). With the exception of confirming the genotype as WT male or DE50-MD male, no additional inclusion or exclusion criteria were used when recruiting dogs to the study. Researchers involved in data acquisition and data analysis for this study were not blinded to genotype.

### Blood sampling

Blood samples for this study were collected longitudinally at 3-monthly intervals from the DE50-MD dogs and WT control dogs from 3 to 18 months of age. Blood was collected by jugular venipuncture into plain tubes, allowed to clot and then centrifuged at 500 ***g*** for 10 min at 4°C. Serum was collected and frozen (in 80 µl aliquots) at −80°C until required for analysis.

### Luminex assay – cytokine/chemokine panel for serum samples

A panel of 13 inflammatory proteins was assayed in multiplex in serum samples by Luminex assay [Canine Cytokine/Chemokine 13-Plex Array (CD13), performed commercially by Eve Technologies, Canada]. These included GM-CSF, IFNγ, IL2, IL6, IL7, IL8, IL10, IL15, IL18, IP-10, KC-like, CCL2 and TNFα.

### Principal component analysis

A principal component analysis was performed using the results for serum concentration of all 13 inflammatory panel proteins for each sample in order to summarise the variation in the dataset and identify patterns between the inflammatory panel proteins. The values of the extracted principal components were compared between WT and DE50-MD dogs and those that best distinguished the two genotypes were further investigated as potential biomarkers of the disease.

### Sample size calculations

For all biomarkers identified in the study, sample size calculations were performed to identify the appropriate number of samples required for prospective pre-clinical trials in order to detect a difference between WT and DE50-MD genotypes with sufficient statistical power (power=0.8). Sample size calculations were performed using the GLIMMPSE online software (Kreidler et al., 2013) based on a two-way repeated measures ANOVA model. The required sample sizes for each biomarker were derived based on the means and standard deviations for the biomarker at each of the six timepoints measured (3, 6, 9, 12, 15 and 18 months of age). We calculated the sample size required to detect a specific percentage change in DE50-MD serum concentration of a biomarker towards WT levels; percentage improvements of 25%, 50%, 75% and 100% were selected to span a range of potential outcomes of future pre-clinical trials.

### Muscle biopsy procedure

Muscle biopsy was performed at 3-monthly intervals from 3 to 18 months of age. Muscle samples (∼0.5 cm^3^) were collected by open biopsy from the left vastus lateralis muscle, with dogs under general anaesthesia. Dogs were premedicated with intravenous methadone (0.2 mg/kg, Comfortan, Dechra) 30 min prior to induction of anaesthesia with intravenous propofol (1-4 mg/kg, PropoFlo Plus, Zoetis) to effect and maintained with sevoflurane (1.5-3.5%, SevoFlo, Zoetis) in oxygen administered via a Datex-Ohmeda Aestiva/5 anaesthesia machine. All dogs received intravenous carprofen (2 mg/kg, Rimadyl, Pfizer) for pain relief and cefuroxime antibiotic (20 mg/kg, Zinacef, GSK). A small piece of muscle tissue was trimmed from each sample, placed in a cryovial and snap frozen in liquid nitrogen, and the remaining tissue was mounted on corks in transverse orientation using cryoMbed (Bright Instruments) and frozen in liquid nitrogen-cooled isopentane. Once frozen, all samples were stored at −80°C until use.

### RNA isolation from tissue

Frozen tissue was crushed under liquid nitrogen using a pestle and mortar. RNA was isolated using TRIzol reagent (Invitrogen, 15596026) according to the manufacturer's instructions, with the addition of a 1:1 chloroform extraction step following initial phase separation and using a modified precipitation protocol [isopropanol (1 vol), 3 M sodium acetate pH 5.5 (0.1 vol) and 10 µg/ml glycogen]. RNA samples were analysed for concentration and purity using a NanoDrop1000 spectrophotometer (Thermo Fisher Scientific). Samples with a 260 nm:230 nm absorbance ratio of less than 1.7 underwent a further isopropanol precipitation step.

### Quantitative reverse-transcription PCR

mRNA was reverse transcribed to complementary DNA (cDNA) using the precision nanoScript2 reverse transcription kit (Primerdesign, RT-nanoscript2) using 1600 ng total RNA per reaction, with oligo(dT) and random nonamer priming. The resulting cDNAs were diluted 1:20 to a final concentration of 4 ng/µl (assuming a 1:1 conversion) and stored at −20°C until further use.

Quantitative polymerase chain reactions (qPCR) were performed in duplicate or triplicate, using PrecisionPLUS PCR master mix with SYBR green (Primerdesign, PPLUS-machine type) and 2 µl of diluted cDNA (8 ng assuming a 1:1 conversion from RNA, as described above) per well. Reactions were performed using a CFX384 thermal cycler (Bio-Rad) starting with a 95°C initial denaturation for 3 min, followed by 40 cycles of the following parameters: 95°C, 15 s; 60°C, 20 s; and 72°C, 20 s. Melt curves were performed for all reactions: 60°C to 95°C, by 0.5°C increments every 5 s. Three reference genes that are known to have stable muscle expression across ages and genotypes in the DE50-MD dog colony (Hildyard et al., 2018) were used to normalise the data: ribosomal protein L13a (RPL13A), hypoxanthine phosphoribosyltransferase 1 (HPRT1) and succinate dehydrogenase (SDHA), with primers from the geNorm *Canis familiaris* set (Primerdesign). The primer sequences for these three reference genes are proprietary; however, we provide anchor nucleotides and context length (Table 2).

**Table 2.**
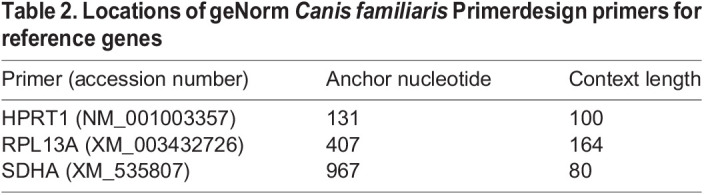
Locations of geNorm *Canis familiaris* Primerdesign primers for reference genes

*CCL2* (NM_001003297) and *CCR2* (XM_005632630) primers were designed using NCBI’s Primer-BLAST software (Ye et al., 2012) and purchased from Eurofins. The following primers were used for quantitative reverse-transcription PCR (RT-qPCR): CCL2 forward, 5′- AAAGAGTCACCAGCAGCAAG-3′; CCL2 reverse, 5′-ATGGCTTTGCAGTTTGGGTT-3′; CCR2 forward, 5′- TGTAAGTCATTCACGGGGCT-3′; and CCR2 reverse, 5′-CTGAGGACTGCAGGAGGAAA-3′.

*CCL2*, *CCR2* and *HPRT1* primers were designed to span introns, precluding amplification of genomic sequence under the PCR conditions used, and a single sharp amplicon peak was seen for all samples. For the primer pairs RPL13A and SDHA that did not span introns, no-reverse-transcriptase controls were tested to establish the levels of genomic DNA within the samples, and the results confirmed that genomic DNA did not significantly contribute to quantification cycle (Cq) values (data available in Hildyard et al., 2018).

Muscle samples for mRNA extraction were not available for every dog and age group for which we had serum (Fig. 9), but *CCL2* and *CCR2* mRNA was quantified in all available corresponding muscle samples.

### Correlation of serum CCL2 concentration with other biomarkers of disease

Work from previous studies showed significantly elevated plasma CK activity (Riddell et al., 2021) and the acid phosphatase-stained fraction of the vastus lateralis muscle (Hildyard et al., 2022) in DE50-MD compared to WT dogs from the same cohort of dogs analysed in the present study. Raw data for these two biomarkers are available at https://doi.org/10.34840/0tfx-4m51 and https://doi.org/10.6084/m9.figshare.20439804.v2, respectively. The interaction between results derived in these earlier studies with the serum CCL2 concentration determined in the present study in samples obtained from the same blood draw (CK activity) or from age-matched vastus lateralis muscle samples (acid phosphatase-stained fraction) was analysed by linear mixed models.

### CK activity

CK activity was quantified in lithium-heparinized plasma using an Ilab600 (Instrumentation Laboratory) clinical chemistry analyser (Riddell et al., 2021).

### Histology

Cork-mounted, frozen vastus lateralis muscle samples were cryosectioned at −25°C at a thickness of 8 µm using a Bright OTF5000 microtome. Sections were mounted onto glass slides (Superfrost), air dried at room temperature for 30 min, then stored frozen at −80°C until use.

#### Acid phosphatase staining

Acid phosphatase staining solution was prepared immediately before use as follows: 3.2 ml of each pararosaniline-HCl (4% pararosaniline and 20% concentrated HCl in distilled water) and 4% sodium nitrite solution were mixed rapidly and incubated at room temperature for 2 min, then added to 200 ml acetate-buffer (350 mM sodium acetate, pH 4.8) supplemented with 4 ml of 1% naphthol AS B1 phosphate (Sigma-Aldrich, N2125) in dimethylformamide. Dilute sodium hydroxide was used to adjust the final pH to 4.8.

Slides were removed from −80°C storage and allowed to warm to room temperature for 30 min before immersion in acid-phosphatase staining solution for 2 h at 37°C. Slides were viewed using an upright bright field microscope (Leica Microsystems, DM4000_BF). Acid phosphatase-positive lysosomes were stained red.

#### H&E staining

Haematoxylin and eosin (H&E) staining was performed as previously described (Dubowitz et al., 2021).

#### Immunohistochemistry

Prior to staining, sections were removed from −80°C and allowed to equilibrate to room temperature for 30 min. Sections were rehydrated in PBS with 0.05% Tween-20 (PBST) for 5 min, and blocked with 5% milk in PBS for 1 h at room temperature. Sections were then incubated with the following combinations of primary antibodies for 1 h at room temperature: goat anti-canine CCL2 IgG (R&D systems, AF1774, 1:20) with rat anti-human perlecan (Millipore, clone A7L6, 1:1000) (Fig. 7), mouse anti-canine CD18 (Bio-Rad, monoclonal IgG1, clone CA1.4E9, MA1-82363, 1:100) (Fig. 8C) or mouse anti-human macrophages (Biorad, monoclonal IgG1, clone MAC387, MCA874GT, 1:100) (Fig. 8D). Both MAC387 anti-human macrophages and A7L6 anti-human perlecan antibodies have confirmed cross-reactivity with canine proteins (Vrolyk et al., 2017; Amoasii et al., 2018). Sections were washed three times with PBST, before incubation with the following corresponding fluorescently labelled secondary antibodies for 1 h at room temperature (protected from light): rabbit anti-rat Alexa Fluor 488 (Invitrogen, A-21210, 1:1000, against the rat anti-human perlecan antibody) or rabbit anti-mouse Alexa Fluor 488 (Invitrogen, A-11059, 1:1000, against the MAC387 and anti-CD18 antibodies), and rabbit anti-goat Alexa Fluor 594 (Invitrogen, A-11080, 1:1000, against the anti-CCL2 antibody). Sections were washed three times with PBST, with Hoechst 33342 (Invitrogen, H3570) dye at 1:1000 added to the first wash. Control sections without primary antibody incubation were also examined. Slides were imaged using an upright fluorescence microscope (Leica Microsystems, DM4000_Fluor).

### Statistical analysis

Data were tested for normality by the Shapiro–Wilk test and, where appropriate, data were log transformed for normalisation. *P*-values of less than 0.05 were considered statistically significant. Linear mixed models were used to evaluate effects of genotype, age and their interaction for serum concentration of the Luminex cytokine/chemokine panel proteins. In some cases, results for the Luminex panel proteins were below the lower limit of quantification. In these cases, samples were assigned the lowest value obtained within the dataset for that particular analyte for the purposes of data analysis (Table S1). Linear mixed models were also used to evaluate interactions between serum CCL2 concentration and age-matched muscle sample *CCL2* mRNA levels, plasma CK activity and acid phosphatase-stained fraction in muscle sections, and the interaction between *CCL2* mRNA and *CCR2* mRNA levels in muscle samples. Post hoc analysis was performed using Tukey's multiple comparisons. A principal component analysis was used to reduce the dimensions of the Luminex cytokine/chemokine panel results and summarise the variation observed within the dataset. Linear mixed modelling, post hoc analyses and the principal component analysis were performed using IBM SPSS Statistics Version 28 and graphs were produced using GraphPad Prism 8.0. Sample size calculations were performed using GLIMMPSE online software (Kreidler et al., 2013), using a two-way repeated measures ANOVA model and a desired power of 0.8.

## Supplementary Material

10.1242/dmm.049394_sup1 Supplementary information
