# Supplementary material for: Serum inflammatory cytokines as disease biomarkers in the DE50-MD dog model of Duchenne muscular dystrophy
Source: Dis Model Mech. 2022 Dec 9;15(12):dmm049394. doi: 10.1242/dmm.049394 (PMC9789403; doi:10.1242/dmm.049394)
Supplement: Supplementary information [file dmm-15-049394-s1.pdf]

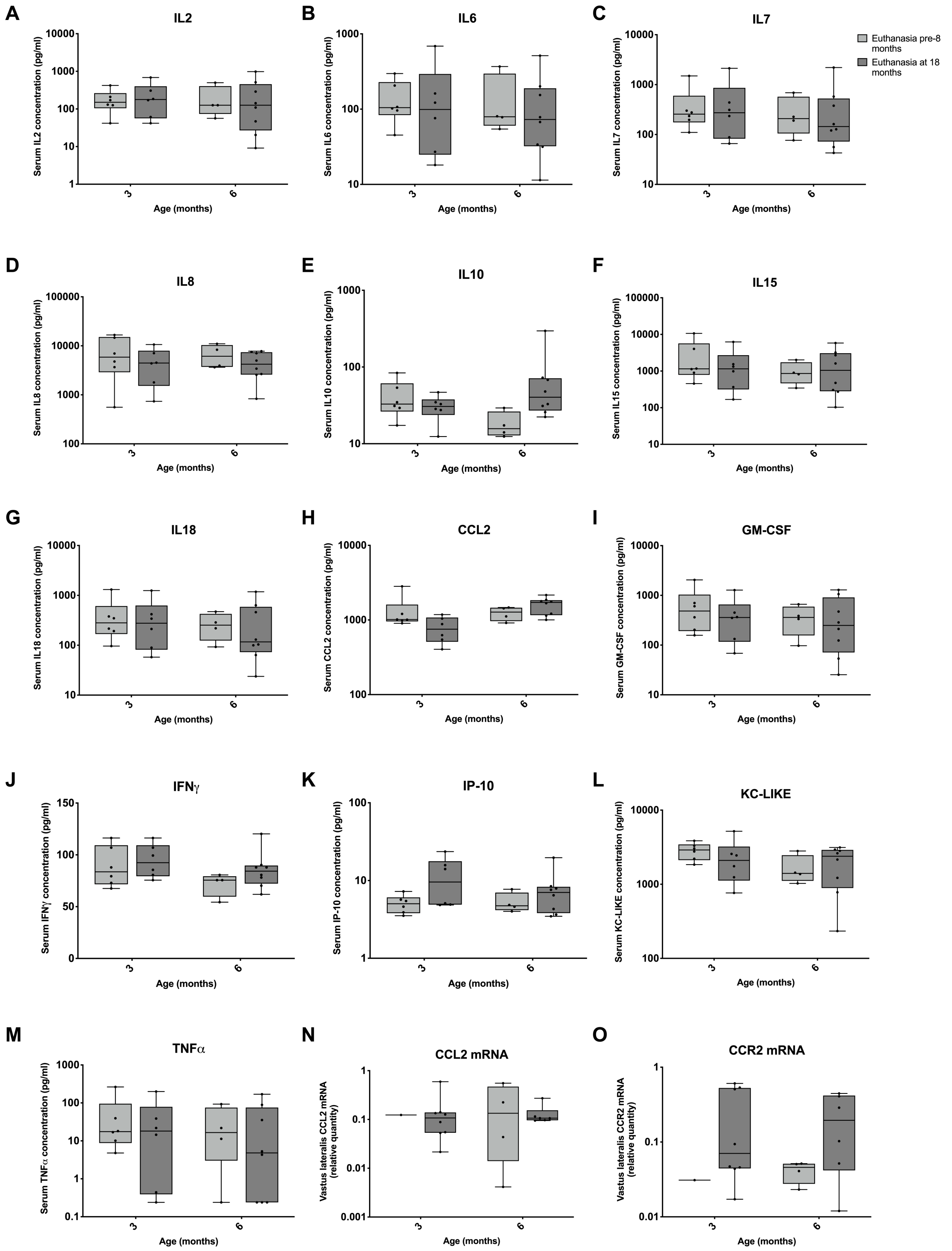

Fig. S1. Comparisons of data for DE50-MD dogs that were euthanased either pre-8 months of age or at 18 months of age. Results for A) interleukin (IL) IL2 , B) IL6, C) IL7, D) IL8, E) IL10, F) IL15, G) IL18, H) CCL2, I) GM-CSF, J) IFNγ K) IP-10, L) KC-LIKE, M) TNFα, N) relative quantity of vastus lateralis muscle CCL2 mRNA and O) relative quantity of vastus

lateralis muscle CCR2 mRNA. DE50-MD dogs that were euthanased pre-8 months of age are shown in light grey (figures A-M: 3 months N=6; 6 months N=4; figures N and O: 3 months N=1, 6 months N=4); DE50-MD dogs that were euthanased at 18 months are shown in dark grey (figures A-M: 3 months N=6; 6 months N=8; figures N and O: 3 months N=8, 6 months N=6). Age is displayed in months. Boxes extend from the 25th to 75th percentile, with a line within the box at the median value. Each point represents an individual sample, and whiskers show the minimum and maximum results for that age-group. There were no significant differences between the 2 groups of DE50-MD dogs in any of the 15 parameters analysed (linear mixed model analyses, P>0.05).

Disease Models & Mechanisms • Supplementary information

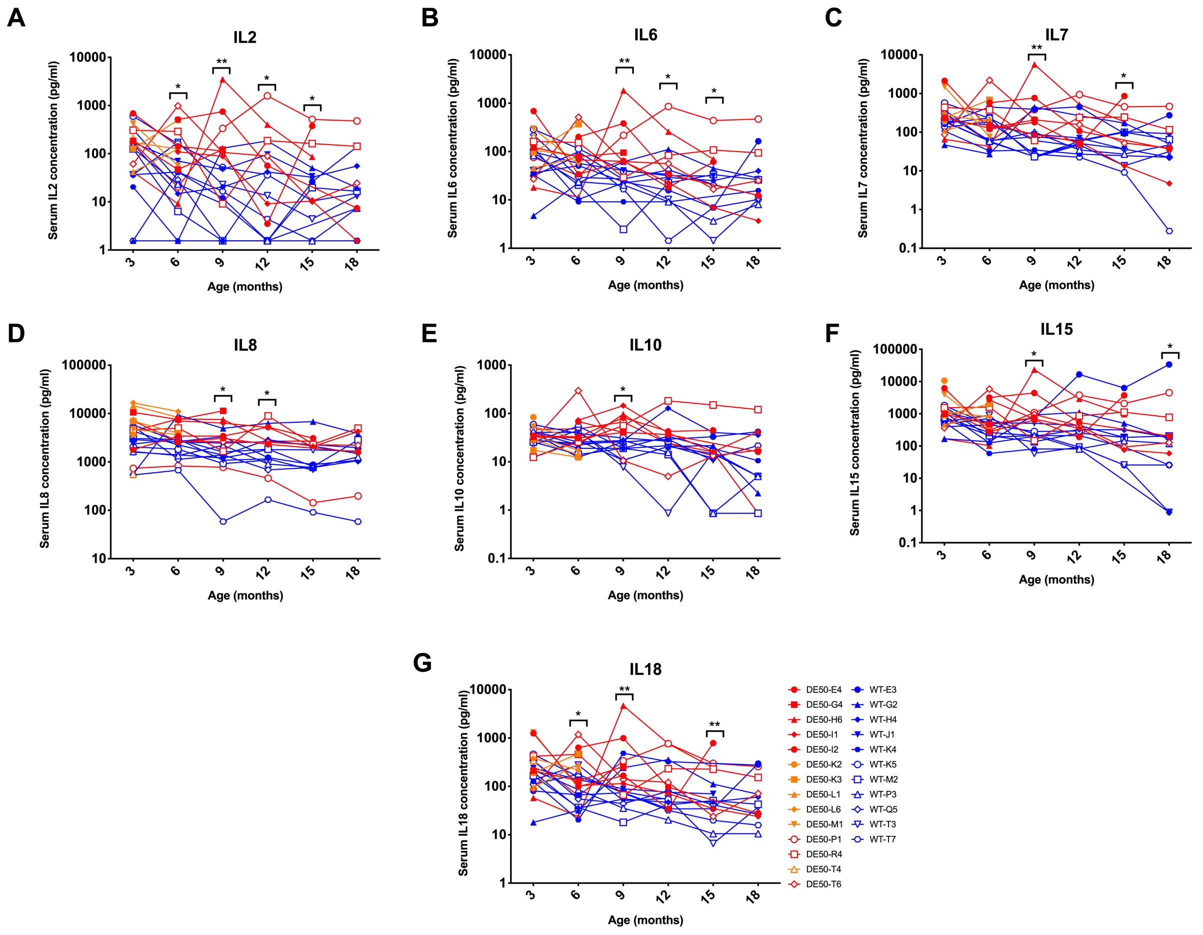

Fig. S2. Serum interleukin concentrations in DE50-MD and WT dogs. Comparison of Luminex assay results for individual DE50-MD and WT dog serum concentrations for interleukins (IL) (A) IL2; (B) IL6; (C) IL7; (D) IL8; (E) IL10; (F) IL15 and (G) IL18. Dogs were studied longitudinally between 3 and 18 months of age. Month 3: WT N=11, DE50-MD N=12; month 6: WT=11, DE50-MD N=12; month 9: WT N=11, DE50-MD N=8; month 12: WT N=11, DE50-MD N=7; month 15: WT N=9, DE50-MD N=6; month 18: WT N=9, DE50-MD N=5. Dog ID is presented in the legend; DE50-MD dogs that completed the 18 month study are labelled red, DE50-MD dogs that were euthanised prior to the end of the study are labelled orange, WT dogs are labeled blue. Asterisks indicate significance level based on linear mixed model analysis, adjusted for repeated measures: *P<0.05, **P<0.01.

Disease Models & Mechanisms • Supplementary information

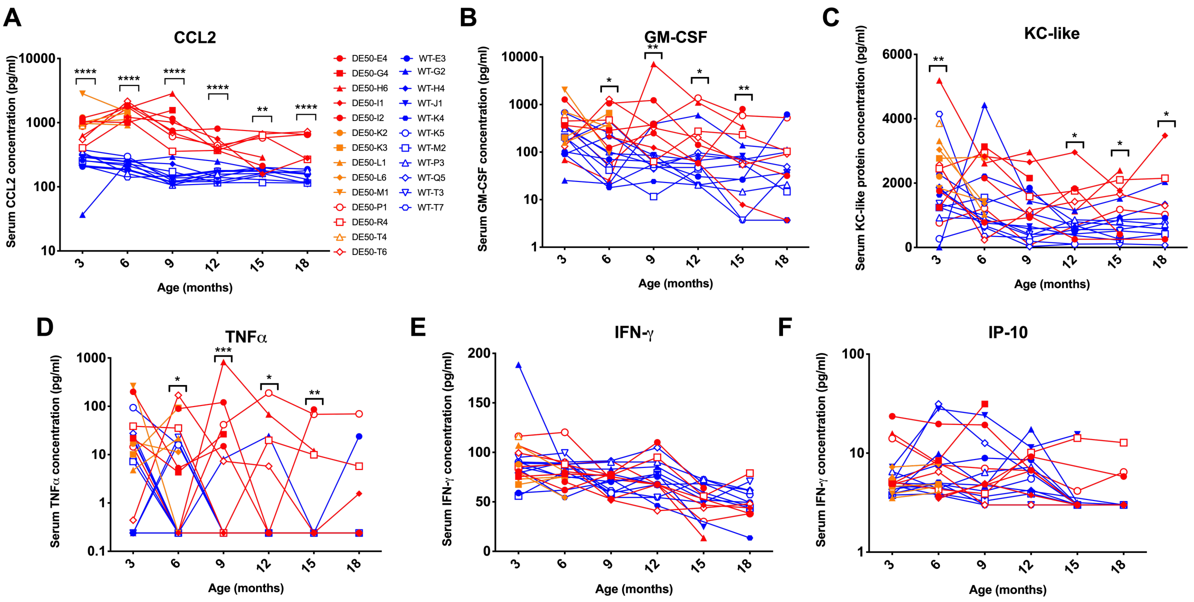

Fig. S3. Serum inflammatory cytokine concentrations in DE50-MD and WT dogs. Comparison of Luminex assay results for individual DE50-MD and WT dog serum concentrations for (A) CCL2; (B) GM-CSF; (C) KC-like protein; (D) TNFα; (E) IFN-γ and (F) IP10. Dogs were studied longitudinally between 3 and 18 months of age. Month 3: WT N=11, DE50-MD N=12; month 6: WT=11, DE50-MD N=12; month 9: WT N=11, DE50-MD N=8; month 12: WT N=11, DE50-MD N=7; month 15: WT N=9, DE50-MD N=6; month 18: WT N=9, DE50-MD N=5. Dog ID is presented in the legend; DE50-MD dogs that completed the 18 month study are labelled red, DE50-MD dogs that were euthanised prior to the end of the study are labelled orange, WT dogs are labeled blue. Concentrations varied over orders of magnitudes between and/or within groups for some of the cytokines measured, therefore a logarithmic scale was used on the y-axis to better display the data for graphs A, B, D and F. Asterisks indicate significance level based on linear mixed model analysis, adjusted for repeated measures: * P<0.05, ** P<0.01, *** P<0.001, **** P<0.0001.

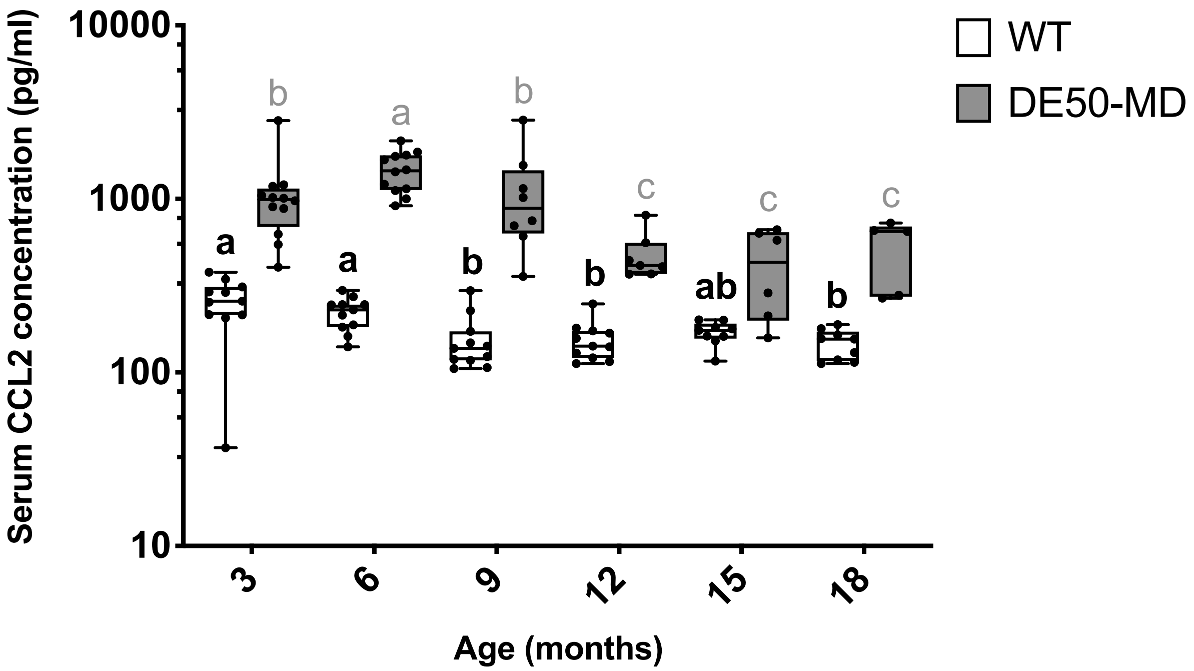

Fig. S4. Effect of age on serum CCL2 concentration in DE50-MD and WT dogs. Boxes extend from the 25th to 75th percentile, with a line within the box at the median value. Each point represents an individual DE50-MD (grey) or WT dog (white) studied longitudinally between 3 and 18 months of age, and whiskers show the minimum and maximum results for that age-group. Month 3: WT N=11, DE50-MD N=12; month 6: WT=11, DE50-MD N=12; month 9: WT N=11, DE50-MD N=8; month 12: WT N=11, DE50-MD N=7; month 15: WT N=9, DE50-MD N=6; month 18: WT N=9, DE50-MD N=5. Letters a, b and c denote statistically significant differences (P<0.05) in the mean within either the DE50-MD (grey letters) or WT (black letters) genotypes: means sharing a letter are not significantly different within each genotype group.

Disease Models & Mechanisms • Supplementary information

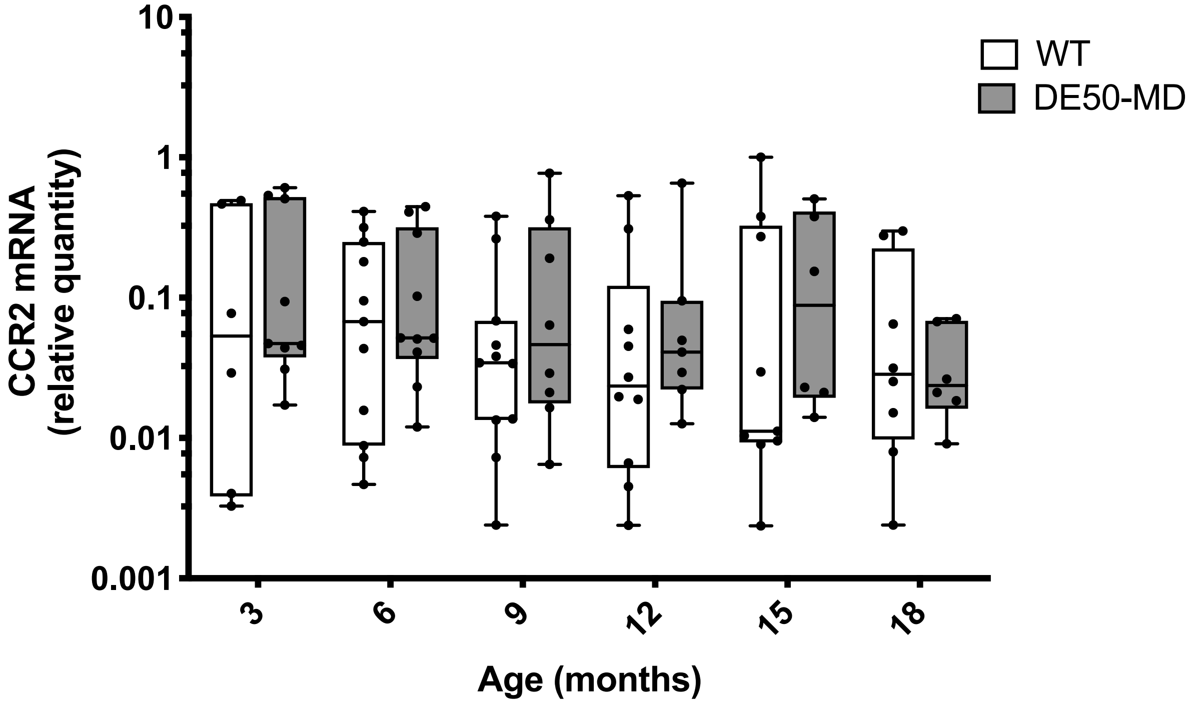

Fig. S5. RT-qPCR of CCR2 mRNA in vastus lateralis muscle of DE50-MD and WT dogs. Results were normalised to the expression of 3 reference genes: RPL13a, HPRT1 and SDHA. Boxes extend from the 25th to 75th percentile, with a line within the box at the median value. Each point represents an individual DE50-MD (grey) or WT dog (white), and whiskers show the minimum and maximum results for that age-group. Month 3: WT N=6, DE50-MD N=9; month 6: WT=11, DE50-MD N=10; month 9: WT N=11, DE50-MD N=8; month 12: WT N=10, DE50-MD N=7; month 15: WT N=9, DE50-MD N=6; month 18: WT N=8, DE50-MD N=6. Analysed by linear mixed model analysis, adjusted for repeated measures.

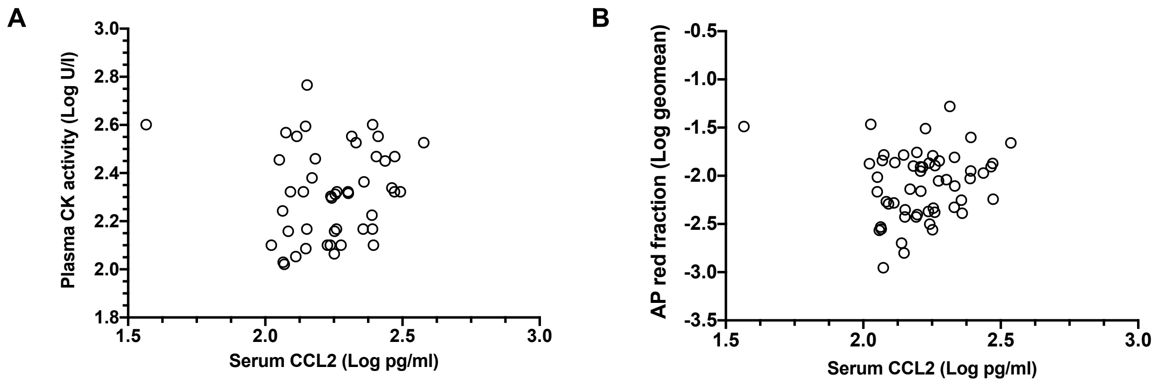

Fig. S6. Relationship between serum CCL2 protein concentration and other potential biomarkers of dystrophinopathy in WT dogs. Graph (A) Relationship between serum CCL2 and plasma CK activity. No relationship was found between the two variables in WT dogs (P=0.99). N=46 samples, from a total of 11 dogs studied longitudinally between 3 and 18 months of age (month 3: N=7; month 6: N=9; month 9: N=10; month 12: N=9; month 15: N=7; month 18: N=4). Graph (B) relationship between serum CCL2 and vastus lateralis muscle acid phosphatase red fraction. No correlation was found between the two variables within WT dogs (P=0.66). N=55 samples from a total of 11 dogs (month 3: N=4; month 6: N=11; month 9: N=11; month 12: N=10; month 15: N=8; month 18: N=9). Linear regressions calculated based on linear mixed model analysis, accounting for repeated measures.

Disease Models & Mechanisms • Supplementary information

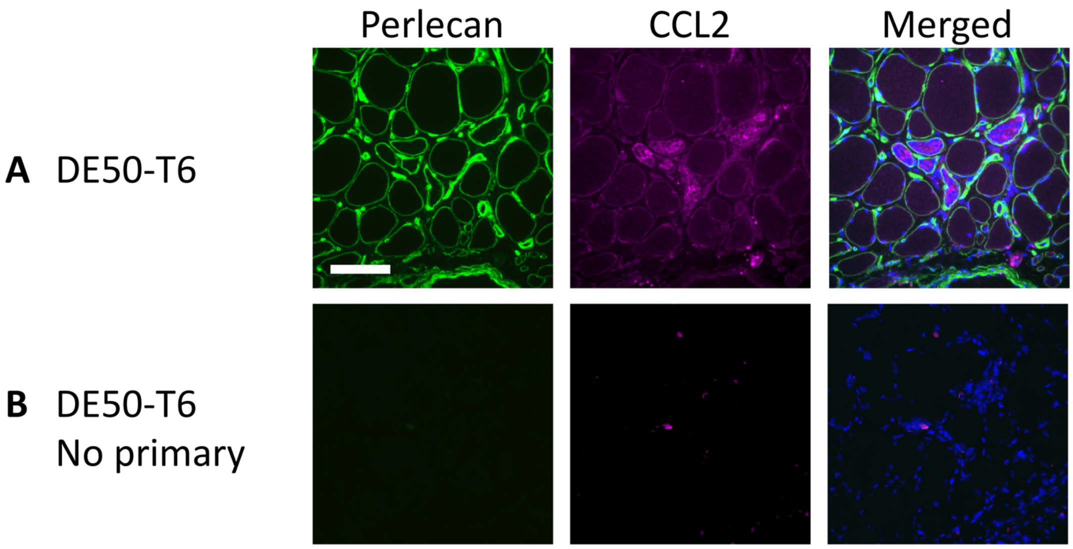

Fig. S7. Immunohistochemistry showing CCL2 protein in vastus lateralis muscle sections. Vastus lateralis sections from an 18 month old DE50-MD dog (dog ID: DE50-T6). (A) Section labelled with primary antibodies to perlecan (green) and CCL2 (magenta), and nuclei labelled with Hoechst (blue). (B) A no-primary antibody control was performed using a serial section of muscle from dog DE50-T6 (scale bar: 100µm).

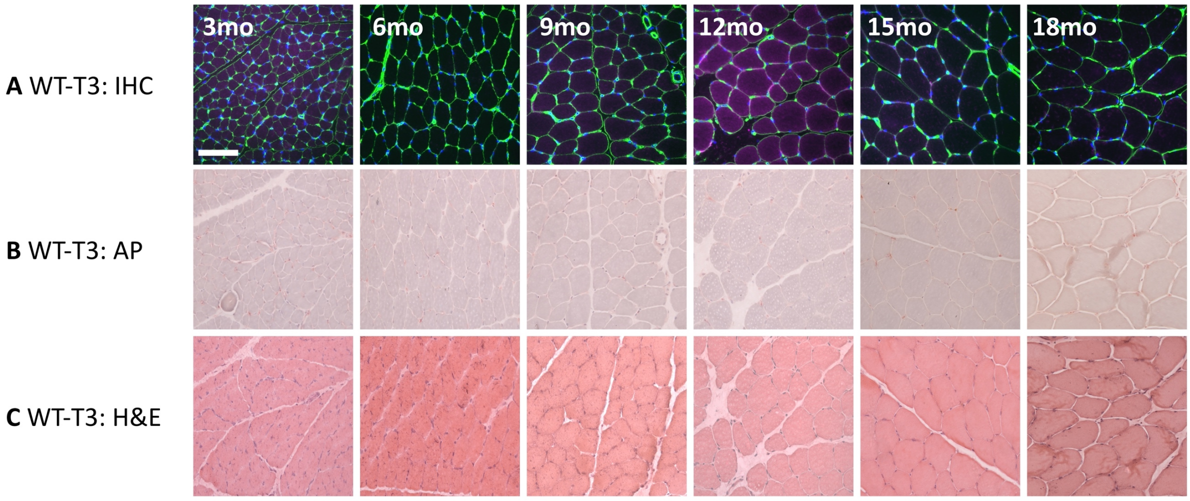

Fig. S8. Histology of the vastus lateralis muscle from a WT dog from 3 to 18 months of age. (A) Immunohistochemistry of vastus lateralis muscle from a wild-type dog (dog ID WT-T3). Each section was labelled with antibodies to perlecan (green) and CCL2 (magenta), and nuclei were labelled with Hoechst (blue). Serial sections for each timepoint were stained for (B) acid phosphatase (AP) and with (C) Haematoxylin and Eosin (H&E) (scale bar for all images: 100µm).

Table S1. Luminex inflammatory panel raw data. In the case that a sample was below the lower limit of quantification (OOR < : out of range), that sample was assigned the lowest value obtained in the dataset for that particular analyte for data analysis purposes. This was necessary for GM-CSF (lowest value: 3.7 pg/ml), IP-10 (3 pg/ml), IL2 (1.55 pg/ml), IL10 (0.86 pg/ml), IL15 (0.88 pg/ml) and TNFα (0.24 pg/ml).

Genotype	ID	Age (months)	IL-2	IL-6	IL-7	IL-8	IL-10	IL-15	IL-18	CCL2	GM-CSF	IFNγ	IP-10	KC-like	TNFα
WT	WT-E5	3	36.63	36.06	164.87	5857.79	38.57	395.46	80.84	206.51	101.57	58.95	5.45	2268.05	OOR<
WT	WT-E5	6	41.78	50.98	446.29	2644.06	12.42	455.76	66.60	182.33	70.26	61.90	7.60	1362.51	OOR<
WT	WT-E5	9	11.97	23.66	390.24	3331.42	25.78	737.29	74.36	117.26	63.32	70.28	8.91	1849.70	OOR<
WT	WT-E5	12	OOR<	15.84	451.11	1227.82	29.28	16629.31	34.20	168.38	30.59	75.56	8.55	508.78	OOR<
WT	WT-E5	15	26.05	6.98	91.73	882.31	32.98	6302.39	34.63	200.61	26.40	51.96	OOR<	856.17	OOR<
WT	WT-E5	18	139.16	163.75	274.55	1080.55	41.85	33701.81	296.24	188.87	618.50	49.36	OOR<	916.94	23.89
WT	WT-G2	3	OOR<	4.69	47.02	559.45	40.56	168.01	18.05	36.81	25.40	188.61	3.77	9.56	OOR<
WT	WT-G2	6	OOR<	22.54	27.08	9310.11	40.56	135.65	31.82	246.05	21.59	83.11	9.87	4430.97	OOR<
WT	WT-G2	9	129.97	61.48	441.46	4940.34	10.78	921.17	242.93	295.74	392.43	83.11	4.61	1438.17	8.18
WT	WT-G2	12	184.21	110.77	266.09	6302.96	20.68	1107.42	358.17	247.98	593.95	85.54	17.40	1147.52	24.44
WT	WT-G2	15	50.46	44.84	172.34	6821.84	21.62	505.94	111.35	181.80	139.47	59.34	3.16	1523.67	OOR<
WT	WT-G2	18	19.71	28.22	49.70	3852.75	2.24	165.36	68.67	130.29	113.88	45.33	OOR<	2039.51	OOR<
WT	WT-H4	2	158.97	120.59	295.86	4316.35	40.56	1128.25	197.48	310.63	215.29	90.28	4.61	1806.72	20.53
WT	WT-H4	6	14.79	10.26	60.87	2243.56	22.36	124.77	35.41	245.78	20.33	74.26	4.49	840.04	OOR<
WT	WT-H4	9	20.39	22.54	76.53	1383.96	27.52	199.95	54.95	227.31	56.71	78.12	3.65	578.21	OOR<
WT	WT-H4	12	41.78	29.28	60.87	2880.75	128.44	324.47	46.80	141.68	85.20	67.55	4.25	641.82	OOR<
WT	WT-H4	15	32.48	25.08	103.26	2124.88	40.35	317.23	48.19	200.29	69.88	68.48	OOR<	953.08	OOR<
WT	WT-H4	18	55.43	39.67	91.73	1487.83	35.89	184.88	60.46	155.72	99.66	49.36	OOR<	1361.47	OOR<
WT	WT-J1	3	169.44	98.57	199.60	2341.88	27.52	1128.25	239.51	289.43	355.58	85.54	7.24	1247.49	22.24
WT	WT-J1	6	70.38	56.80	151.58	2978.65	24.06	535.84	137.80	244.67	93.22	75.56	27.97	760.06	OOR<
WT	WT-J1	9	46.79	40.62	88.07	1081.87	25.78	505.83	89.27	105.24	85.20	70.28	24.09	652.23	OOR<
WT	WT-J1	12	96.61	31.53	71.09	1160.17	29.28	435.70	75.96	112.39	66.75	78.12	11.42	373.93	OOR<
WT	WT-J1	15	34.09	31.35	36.63	671.13	10.53	184.88	70.04	152.17	82.90	24.62	15.49	222.64	OOR<
WT	WT-K4	3	20.39	32.66	107.03	2897.20	34.74	555.86	126.19	257.84	91.18	83.11	3.89	1628.87	OOR<
WT	WT-K4	6	OOR<	9.15	32.73	2591.67	19.01	58.77	20.30	214.11	17.82	54.38	4.97	2218.81	OOR<
WT	WT-K4	9	OOR<	9.15	34.66	2452.37	19.01	80.88	483.71	137.74	24.12	72.95	4.73	1708.31	OOR<
WT	WT-K4	12	OOR<	9.15	27.08	2623.18	33.80	80.88	325.12	140.61	20.33	46.21	4.13	799.90	OOR<
WT	WT-K4	18	7.37	15.58	22.51	1978.34	10.53	OOR<	276.46	118.21	36.97	13.52	OOR<	580.55	OOR<
WT	WT-K5	3	599.17	289.69	564.06	2039.31	58.61	1797.66	459.95	378.08	672.02	90.28	4.97	270.94	93.76
WT	WT-K5	6	158.97	114.45	229.35	1832.87	38.57	455.76	156.97	296.53	249.40	89.11	4.85	679.08	15.92
WT	WT-K5	9	51.69	39.48	60.87	922.14	31.06	283.44	77.57	123.55	47.39	58.95	4.13	195.25	OOR<
WT	WT-K5	12	36.63	33.79	42.75	1091.71	26.64	293.73	53.56	129.27	47.39	67.55	5.50	103.92	OOR<
WT	WT-M2	3	125.84	97.36	193.46	3042.85	25.78	696.76	166.37	254.02	207.05	55.93	3.77	1242.06	7.22
WT	WT-M2	6	6.33	20.30	58.45	2913.60	24.06	210.51	36.63	229.01	41.54	80.64	3.89	1553.90	OOR<
WT	WT-M2	9	OOR<	2.46	23.41	1607.19	19.01	135.65	18.05	172.56	11.62	58.95	3.29	1058.08	OOR<
WT	WT-M2	12	OOR<	22.54	58.45	1832.87	14.06	252.38	41.62	115.65	44.43	79.38	3.89	691.61	OOR<
WT	WT-M2	15	19.71	33.44	99.35	801.08	OOR<	184.88	50.91	116.23	56.32	55.72	OOR<	255.03	OOR<
WT	WT-M2	18	16.57	26.13	64.36	2856.30	OOR<	205.07	42.75	114.41	14.63	43.94	OOR<	422.17	OOR<
WT	WT-P3	3	127.91	82.86	187.25	1638.24	42.60	767.77	218.74	344.34	312.14	89.11	6.52	927.19	9.63
WT	WT-P3	6	23.17	23.66	58.45	1355.92	25.78	262.76	85.85	161.52	101.57	87.93	4.37	890.69	OOR<
WT	WT-P3	9	9.15	20.30	100.51	2205.08	27.52	210.51	35.41	106.53	55.11	90.28	6.4	370.32	OOR<
WT	WT-P3	12	OOR<	9.15	34.66	906.96	15.70	80.88	20.30	157.19	20.33	90.28	7.24	860.92	OOR<
WT	WT-P3	15	1.55	3.68	27.09	769.59	OOR<	146.49	10.53	161.85	14.63	72.80	OOR<	822.05	OOR<
WT	WT-P3	18	7.37	8.07	46.96	1271.07	5.01	119.26	10.53	178.87	20.72	61.69	OOR<	718.20	OOR<
WT	WT-Q5	3	315.94	223.07	372.88	5530.43	12.42	1679.09	340.94	214.69	376.75	79.38	4.97	1866.64	27.63
WT	WT-Q5	6	28.64	29.28	56.07	1126.04	27.52	189.35	54.95	187.89	50.42	90.28	31.38	626.61	OOR<
WT	WT-Q5	9	OOR<	27.03	23.41	1402.57	10.78	210.51	44.18	118.85	44.43	91.45	12.62	28.26	OOR<
WT	WT-Q5	12	36.63	41.76	51.45	685.71	20.68	210.51	79.19	121.22	97.35	104.82	6.22	104.43	OOR<
WT	WT-Q5	15	10.39	20.87	36.63	769.59	14.67	93.01	41.40	161.45	74.27	68.48	OOR<	112.09	OOR<
WT	WT-Q5	18	15.01	12.38	22.51	1062.45	5.01	25.71	26.54	112.54	49.27	61.69	3.00	75.30	OOR<
WT	WT-T3	3	36.63	40.62	168.13	1598.28	22.36	575.90	135.87	291.54	168.75	94.89	4.73	1366.26	OOR<
WT	WT-T3	6	171.54	98.57	235.13	1397.93	44.69	900.62	276.50	273.49	450.32	99.38	3.77	997.40	22.72
WT	WT-T3	9	22.87	26.13	22.51	1207.74	7.77	59.03	50.91	142.03	60.91	54.48	OOR<	409.46	OOR<
WT	WT-T3	12	13.46	10.23	46.96	1798.33	0.86	93.01	56.36	173.45	56.32	54.48	OOR<	420.36	OOR<
WT	WT-T3	15	4.41	1.43	13.57	1784.14	16.06	25.71	6.59	174.55	OOR<	49.36	OOR<	514.47	OOR<
WT	WT-T3	18	13.46	10.23	31.78	1672.28	5.01	0.88	37.33	164.36	36.97	70.65	OOR<	420.36	OOR<
WT	WT-T7	3	OOR<	31.53	134.41	538.09	25.78	475.80	110.93	215.27	91.18	83.11	4.31	4147.52	OOR<

WT	WT-T7	6	41.78	82.86	187.25	675.26	54.89	717.01	177.49	140.25	215.29	90.28	5.03	357.63	17.37
WT	WT-T7	9	16.57	16.64	27.09	58.46	18.83	236.70	72.78	148.05	87.15	61.69	OOR<	309.85	OOR<
WT	WT-T7	12	4.41	1.43	22.51	166.29	21.62	93.01	31.93	179.81	20.72	54.48	OOR<	553.72	OOR<
WT	WT-T7	15	1.55	6.98	9.15	90.99	11.91	25.71	19.84	178.51	OOR<	72.80	OOR<	559.45	OOR<
WT	WT-T7	18	1.55	10.23	0.28	58.46	21.62	25.71	15.84	156.55	OOR<	56.94	OOR<	762.84	OOR<
DE50-MD	DE50-E4	6	512.62	200.84	576.19	2559.93	68.06	3201.84	632.23	1860.41	1057.97	70.28	3.65	2153.05	88.99
DE50-MD	DE50-E4	9	746.20	380.71	768.38	3087.32	40.56	4413.89	991.96	747.76	1229.49	52.80	4.85	979.66	120.71
DE50-MD	DE50-E4	12	56.49	33.79	107.03	2311.89	36.63	535.84	96.28	805.78	141.94	67.55	9.21	254.65	OOR<
DE50-MD	DE50-E4	18	7.37	12.38	36.63	1616.88	16.06	205.07	27.89	649.86	31.79	38.12	5.81	255.69	OOR<
DE50-MD	DE50-G4	3	184.21	121.82	235.13	10610.32	32.88	982.99	211.71	1045.73	366.20	75.56	4.91	1242.06	21.75
DE50-MD	DE50-G4	6	46.79	78.07	127.47	7536.93	31.06	475.80	130.05	1002.67	286.01	78.12	8.43	3135.30	4.32
DE50-MD	DE50-G4	9	121.71	94.93	208.69	11382.15	40.56	757.60	256.49	1563.63	375.00	76.85	31.38	2157.18	26.40
DE50-MD	DE50-H6	3	41.78	18.07	65.88	4386.30	28.39	168.01	57.78	625.10	68.49	106.96	15.78	5189.33	OOR<
DE50-MD	DE50-H6	6	9.15	11.38	42.75	7779.61	22.36	102.89	23.70	1678.59	25.40	87.93	7.9	2619.01	OOR<
DE50-MD	DE50-H6	9	3506.92	1830.50	5643.07	7536.93	96.95	23482.31	4684.77	2847.88	7100.11	91.45	4.73	2965.97	826.44
DE50-MD	DE50-H6	12	401.20	257.63	518.32	2688.96	24.06	2921.15	763.67	413.05	1105.16	67.55	6.64	1124.04	67.90
DE50-MD	DE50-H6	15	85.70	69.49	187.57	2699.90	13.29	971.58	242.63	287.02	343.99	13.52	OOR<	2394.62	12.02
DE50-MD	DE50-I1	6	109.25	67.37	161.58	7053.04	72.48	2698.04	103.51	1214.46	211.18	80.64	3.47	2831.63	OOR<
DE50-MD	DE50-I1	9	88.04	62.66	88.07	6352.55	145.31	686.64	114.70	1016.93	123.67	75.56	4.97	2650.64	OOR<
DE50-MD	DE50-I1	12	9.15	22.54	56.07	5179.43	39.56	355.00	71.21	558.95	59.97	67.55	3.77	2956.33	OOR<
DE50-MD	DE50-I1	15	10.39	6.98	13.57	2092.00	16.06	75.91	34.63	158.21	7.78	46.70	OOR<	1764.72	OOR<
DE50-MD	DE50-I1	18	OOR<	3.68	4.73	4254.68	41.85	59.03	23.86	278.97	3.70	43.94	OOR<	3475.91	1.56
DE50-MD	DE50-I2	3	685.91	686.18	2125.10	1790.35	34.74	6234.79	1247.82	1180.68	1274.84	80.64	23.55	1753.39	199.42
DE50-MD	DE50-I2	6	142.35	33.79	120.55	3424.33	32.88	273.12	99.87	1787.95	123.67	61.90	19.67	778.92	5.29
DE50-MD	DE50-I2	9	113.41	63.83	180.96	3439.18	82.13	646.26	166.37	1147.76	249.40	78.12	19.25	928.03	14.95
DE50-MD	DE50-I2	12	3.51	18.07	47.02	5247.73	42.60	189.35	34.20	368.73	58.33	110.12	6.7	1834.00	OOR<
DE50-MD	DE50-I2	15	376.06	61.30	858.12	3103.24	44.89	3743.34	785.30	212.06	802.70	64.00	OOR<	408.85	85.97
DE50-MD	DE50-K2	3	129.97	102.22	196.54	3671.81	83.85	10661.34	215.23	1205.80	362.67	87.93	5.45	2207.64	16.89
DE50-MD	DE50-K3	3	125.84	96.14	235.13	6978.32	17.35	900.62	192.08	1009.34	202.89	67.55	4.61	2768.58	10.12
DE50-MD	DE50-K3	6	498.26	368.43	688.13	3948.34	12.42	2014.81	471.85	1433.51	662.51	75.56	4.85	2808.65	93.16
DE50-MD	DE50-L1	3	41.78	45.21	110.36	14646.90	29.28	455.76	96.28	976.52	157.72	106.96	3.53	3310.95	4.80
DE50-MD	DE50-L1	6	125.84	78.07	226.44	8317.49	14.06	900.62	286.38	913.50	387.22	80.64	4.01	1026.92	21.75
DE50-MD	DE50-L2	3	171.54	107.10	277.04	16657.98	34.74	1128.25	378.30	1016.93	700.48	79.38	7.24	3041.93	18.10
DE50-MD	DE50-L2	6	125.84	80.47	190.36	11017.80	17.35	818.71	218.74	1117.24	334.05	54.38	7.72	1371.25	11.32
DE50-MD	DE50-M1	3	422.31	297.08	1492.19	4781.69	53.69	4021.31	1308.98	2822.61	2046.46	72.95	3.89	1840.61	263.87
DE50-MD	DE50-M1	6	56.49	54.47	76.53	3646.08	29.28	344.84	92.75	1470.02	97.35	75.56	4.61	1433.18	OOR<
DE50-MD	DE50-P1	3	171.54	75.69	311.69	737.75	46.85	1316.99	340.94	880.93	352.01	116.28	14.05	764.35	14.47
DE50-MD	DE50-P1	6	20.39	31.53	56.07	830.54	47.95	304.00	63.60	1761.29	53.53	120.29	7.6	1219.08	OOR<
DE50-MD	DE50-P1	9	334.15	216.90	382.82	773.97	66.63	1086.61	337.79	608.57	385.48	72.95	7	71.76	41.75
DE50-MD	DE50-P1	12	1587.15	852.28	937.19	460.23	24.06	3792.05	765.16	442.62	1368.47	67.55	6.64	666.10	188.09
DE50-MD	DE50-P1	15	515.23	439.40	452.68	143.41	16.06	2081.69	299.07	577.64	586.75	30.07	4.15	1179.09	68.37
DE50-MD	DE50-P1	18	477.30	468.87	464.16	198.04	0.86	4491.36	256.71	657.57	526.54	38.12	6.45	1023.72	69.72
DE50-MD	DE50-R4	3	306.89	161.32	439.04	4526.93	12.42	1539.82	419.46	404.51	450.32	85.54	5.09	2448.70	38.67
DE50-MD	DE50-R4	6	288.90	153.90	377.86	5000.66	25.78	1614.69	459.95	1144.20	472.00	87.93	4.31	2935.38	35.37
DE50-MD	DE50-R4	9	9.15	29.28	60.87	1660.29	56.11	146.49	66.60	357.32	63.32	76.85	3.89	1584.15	OOR<
DE50-MD	DE50-R4	12	186.33	82.86	240.87	8940.22	182.42	859.61	232.64	369.23	274.60	94.89	10.11	1759.18	19.80
DE50-MD	DE50-R4	15	161.97	107.14	244.55	2304.86	149.38	1119.38	227.16	634.72	232.09	55.72	14.21	2101.46	9.93
DE50-MD	DE50-R4	18	142.66	93.94	115.35	4981.01	120.38	778.50	153.00	268.03	103.76	79.03	12.75	2150.94	5.75
DE50-MD	DE50-T4	3	209.84	205.78	301.17	559.45	31.06	1170.00	347.23	902.21	605.16	116.28	5.68	3858.80	39.43
DE50-MD	DE50-T6	3	61.20	27.03	88.07	7075.82	27.52	365.14	89.27	545.75	132.79	99.38	4.85	2566.62	0.44
DE50-MD	DE50-T6	6	983.23	512.65	2200.81	2651.00	295.95	5815.05	1180.81	2157.13	1286.93	90.28	6.46	233.58	169.19
DE50-MD	DE50-T6	9	106.22	62.32	209.59	2623.11	10.53	957.98	139.08	699.62	323.34	51.96	OOR<	1152.73	7.32
DE50-MD	DE50-T6	12	89.11	56.17	156.61	2551.31	5.01	421.15	121.04	407.27	201.35	41.09	OOR<	1423.85	5.75
DE50-MD	DE50-T6	15	10.39	16.64	52.49	2260.49	13.29	93.01	23.86	663.82	53.99	43.94	OOR<	1660.68	OOR<
DE50-MD	DE50-T6	18	24.46	25.08	39.13	2212.50	17.44	128.21	71.41	727.13	91.36	48.04	OOR<	1299.22	OOR<

Table S2. Mean, standard deviation (SD) and range of serum concentration of cytokines in the Luminex panel.

		3 months			6 months			9 months			12 months			15 months			18 months		
		Mean	SD	Range	Mean	SD	Range	Mean	SD	Range	Mean	SD	Range	Mean	SD	Range	Mean	SD	Range
**CCL2**	WT	254.5	90.5	37-378	220.1	48.0	140-297	154.1	58.6	105-296	153.4	39.1	112-248	169.7	26.1	116-201	146.6	28.7	113-189
	DE50-MD	1051.3	610.0	404-2822	1461.7	390.6	913-2157	1123.7	788.5	357-2848	480.8	157.2	369-806	422.2	228.0	158-664	516.3	223.7	268-727
**IL2**	WT	144.9	178.1	<2-599	51.0	60.2	<2-172	28.6	37.7	<2-130	38.2	56.5	<2-184	20.1	17.1	<2-50	30.6	43.6	<2-139
	DE50-MD	212.7	185.1	42-686	243.3	289.3	9-983	628.2	1186.6	9-3506	333.3	569.9	4-1587	193.3	208.1	10-515	130.7	202.1	<2-477
**IL6**	WT	96.2	88.1	5-290	47.2	36.8	9-114	26.3	16.2	2-61	28.7	30.0	<1.43-111	19.4	15.4	<1.43-45	34.9	49.5	8-164
	DE50-MD	162.0	182.8	18-686	139.2	153.2	11-513	342.6	612.4	29-1830	189.0	304.0	18-852	116.8	162.2	7-439	120.8	197.8	4-469
**IL7**	WT	221.3	143.3	47-564	140.3	128.1	27-446	117.2	150.7	23-441	103.0	133.8	22-451	65.5	54.4	9-172	67.2	82.2	0.3-274
	DE50-MD	489.8	639.2	66-2125	403.7	602.4	43-2201	942.8	1912.4	61-5643	294.7	326.2	47-937	301.5	314.3	14-858	132.0	190.1	5-464
**IL8**	WT	2760.0	1813.1	538-5858	2642.7	2341.7	675-9310	1872.1	1333.3	58-4940	1879.7	1673.3	166-6303	1635.1	2038.3	91-6822	1702.2	1104.7	58-3853
	DE50-MD	6368.6	5194.3	559-16658	5313.8	2995.3	831-11018	4606.9	3556.0	774-11382	3911.4	2783.8	460-8940	2100.7	1025.9	143-3103	2652.6	1954.4	198-4981
**IL10**	WT	33.6	12.5	12-59	29.4	13.5	12-55	20.3	7.9	8-31	31.0	33.6	<0.9-128	16.6	13.3	<0.9-40	14.2	15.3	<0.9-42
	DE50-MD	36.1	18.7	12-84	55.8	78.1	12-296	67.3	41.3	11-145	50.6	59.5	5-182	42.2	53.9	13-149	39.3	47.6	<0.9-120
**IL15**	WT	851.7	523.8	168-1798	367.9	269.4	59-901	325.5	278.5	59-921	1781.9	4933.1	81-16629	865.1	2044.5	26-6302	3825.5	11203.9	0.9-33701
	DE50-MD	2412.1	3130.3	168-10661	1547.0	1686.8	103-5815	4022.2	7973.7	147-23482	1296.3	1444.3	189-3792	1347.5	1390.2	76-3743	1132.4	1899.2	59-4491
**IL18**	WT	190.4	124.2	18-460	98.2	80.2	20-277	113.1	136.5	18-484	102.1	120.0	20-358	43.7	32.6	7-111	92.8	111.5	11-296
	DE50-MD	408.8	423.2	58-1309	313.6	334.2	24-1181	844.7	1579.7	67-4685	297.7	324.7	34-765	268.8	277.5	24-785	106.6	98.7	24-257
**GM-CSF**	WT	237.9	184.4	25-672	121.1	134.0	18-450	84.4	104.6	12-392	98.5	166.4	20-594	52.4	44.9	4-139	110.5	194.1	4-619
	DE50-MD	560.0	571.1	68-2046	416.5	401.3	25-1287	1231.2	2398.5	63-7100	458.5	542.4	58-1368	337.9	309.4	8-803	151.4	213.7	4-527
**KC-LIKE**	WT	1525.6	1097.5	10-4148	1338.0	1148.4	358-4431	781.6	633.0	28-1850	564.3	314.6	104-1148	646.5	444.6	112-1524	810.8	584.4	75-2040
	DE50-MD	2582.7	1194.7	764-5189	1878.8	982.8	234-3135	1561.3	974.0	72-2966	1431.2	881.3	255-2956	1584.9	708.3	409-2395	1641.1	1229.2	256-3476
**IFNγ**	WT	90.8	34.8	56-189	80.6	13.3	54-99	71.9	12.8	54-91	73.1	17.3	46-105	58.2	15.4	25-73	50.3	16.3	14-71
	DE50-MD	91.3	17.2	68-116	80.3	16.4	54-120	72.1	13.3	52-91	73.8	22.3	42-110	42.3	18.2	14-64	49.5	17.1	38-79
**IP-10**	WT	4.9	1.1	4-7	9.8	10.0	4-31	7.1	6.4	3-24	6.8	4.4	3-17	4.4	4.2	3-15	3.0	<0.00	3-3
	DE50-MD	8.2	6.2	4-24	6.9	4.4	3-20	9.9	10.1	3-31	6.6	2.6	3-10	5.1	4.5	3-14	6.2	4.0	3-13
**TNFα**	WT	16.6	27.6	<0.3-94	5.3	8.8	<0.3-23	1.0	2.4	<0.3-8	2.4	7.3	<0.3-24	0.2	<0.00	<0.3	2.9	7.9	<0.3-24
	DE50-MD	52.4	85.8	<0.3-264	35.9	53.7	<0.3-169	129.8	284.3	<0.3-826	40.3	69.6	<0.3-188	29.5	37.7	<0.3-86	15.5	30.4	<0.3-70
